# Chronic lymphocytic leukemia with *IGH*::*BCL3*‐translocation is characterized by a homogeneous and distinct genetic and epigenetic landscape

**DOI:** 10.1002/hem3.70354

**Published:** 2026-04-29

**Authors:** Cosima Drewes, Cristina López, Juan Emilio Martinez‐Manjón, Nnamdi Okeke, Billy Jebaraj, Susanne Bens, Björn Brändl, Martin J. S. Dyer, Barbara Eichhorst, Anja Fischer, Kirsten Fischer, Sandra Robrecht, Michael Hallek, Selina Glaser, Sina Hillebrecht, Sandrine Jayne, Helene Kretzmer, Anja Mottok, Franz‐Josef Müller, Christof Schneider, Ole Ammerpohl, Coral del Val, Stephan Stilgenbauer, Eugen Tausch, Reiner Siebert

**Affiliations:** ^1^ Institute of Human Genetics, Ulm University and Ulm University Medical Center Ulm Germany; ^2^ Molecular Pathology of Lymphoid Neoplasms Group Institut d'Investigaciones Biomèdiques August Pi i Sunyer (IDIBAPS) Barcelona Spain; ^3^ Hematopathology Section, Pathology Department Hospital Clínic de Barcelona Barcelona Spain; ^4^ Departament de Fonaments Clínics Universitat de Barcelona Barcelona Spain; ^5^ Department of Computer Science and Artificial Intelligence University of Granada, Andalusian Research Institute in Data Science and Computational Intelligence Granada Spain; ^6^ Instituto de Investigación Sanitaria ibs, GRANADA, Hospitales Universitarios de Granada‐Universidad de Granada Granada Spain; ^7^ Division of CLL, Department of Internal Medicine III Ulm University Medical Center Ulm Germany; ^8^ Department of Psychiatry and Psychotherapy Zentrum für Integrative Psychiatrie gGmbH, Universitätsklinikum Schleswig‐Holstein, Campus Kiel Kiel Germany; ^9^ The Ernest and Helen Scott Haematological Research Institute, Leicester Cancer Research Centre University of Leicester Leicester UK; ^10^ Department I of Internal Medicine and German CLL Study Group Center for Integrated Oncology Aachen Bonn Cologne Duesseldorf (CIO ABCD), University of Cologne, Faculty of Medicine and University Hospital of Cologne Cologne Germany; ^11^ Department of Genome Regulation Max Planck Institute for Molecular Genetics Berlin Germany; ^12^ Digital Health Cluster, Hasso Plattner Institute for Digital Engineering, Digital Engineering Faculty University of Potsdam Potsdam Germany; ^13^ German Center for Child and Adolescent Health (DZKJ), Partner Site Ulm Ulm Germany

## Abstract

Approximately 1% of chronic lymphocytic leukemia (CLL) cases harbor a translocation juxtaposing the *immunoglobulin heavy chain* (*IGH*) and *B‐cell lymphoma 3* (*BCL3*) loci. Aiming at comprehensive molecular characterization of *IGH*::*BCL3‐*positive B‐cell neoplasms, we here investigated samples from 84 patients using fluorescence in situ hybridization (FISH), whole‐genome and targeted sequencing, and DNA methylation analyses. Junctional sequences obtained in 27 patients showed breakpoints upstream of *BCL3* in all CLL cases. *IGH* breaks were presumably driven by aberrant class‐switch recombination in 26/27 cases, frequently involving *IGHA* (12/26). Notably, 95% (78/82) of patients carried an unmutated *IGHV* with significant CLL stereotype subset #8 enrichment. Trisomy 12 (61%, 51/83) and mutations affecting *NOTCH1* (32%, 25/79), *BRAF* (14%, 11/79), and *FBXW7* (14%, 11/79) were frequent aberrations. DNA methylation analysis assigned 77% (51/66) of patients with *IGH*::*BCL3‐*translocation with at least 60% tumor cell content to the naive B‐cell‐like group but unraveled a distinct and during follow‐up stable signature resembling in part plasma cell‐like epigenetic features. A binary DNA methylation classifier using 20 CpGs could distinguish *IGH*::*BCL3*‐translocated CLL samples from other CLL subtypes. In an efficacy cohort of 3832 previously untreated patients from GCLLSG trials, *IGH::BCL3* was associated with shorter progression‐free survival (PFS) and overall survival (OS) in 28 patients when treated with chemoimmunotherapy, but not in those receiving venetoclax. Our findings highlight the genetic and epigenetic homogeneity of *IGH*::*BCL3*‐translocated CLL samples and their differences from other types of CLL, suggesting *IGH*::*BCL3* leukemic B‐cell neoplasms to be a biological distinct type within the spectrum of mature lymphatic leukemia/CLL.

## INTRODUCTION

Chronic lymphocytic leukemia (CLL) is the most prevalent leukemia among adults in Western countries.^1^ It is characterized by the gradual accumulation of monoclonal CD5‐positive B cells and a highly variable clinical course.^1^ Approximately 1% of cases diagnosed as CLL, often with atypical features, carry an *immunoglobulin heavy chain* (*IGH*)::*B‐cell lymphoma 3* (*BCL3*)‐translocation.^2^ Although at much lower frequencies with an estimated fraction of 1.5% based on all cytogenetically detectable cases, the respective *IG* light‐chain variants also occur.^3^, ^4^ The translocation juxtaposes the *IGH* locus in chromosome 14q32 with the *BCL3* gene in chromosome 19q13. This leads to *IGH* enhancer hijacking and subsequently deregulated expression of *BCL3*,^5^ which encodes a transcriptional coactivator of the NF‐κB signaling pathway.^6^

The t(14;19)(q32;q13) underlying the *IGH*::*BCL3*‐juxtaposition was first described cytogenetically in three CLL samples in 1985.^7^, ^8^ Since then, more than 300 cases of B‐cell neoplasms with cytogenetic or molecular genetic evidence of an *IGH*::*BCL3*‐translocation have been reported in the literature.^2^, ^4^, ^5^, ^8^, ^9^, ^10^, ^11^, ^12^, ^13^, ^14^, ^15^, ^16^, ^17^, ^18^, ^19^, ^20^, ^21^, ^22^, ^23^, ^24^, ^25^, ^26^ The majority of these cases has been diagnosed as CLL, while a smaller proportion have been classified as marginal zone lymphoma (MZL) or other B‐cell neoplasms.^4^, ^10^, ^16^, ^17^ Mapping the breakpoints on chromosome 19q13 in a small set of B‐cell malignancies suggested that in CLL, those are mostly located 5′ upstream (centromeric) of the *BCL3* gene, whereas in other B‐cell malignancies the breakpoints could also be located 3′ downstream (telomeric) of the *BCL3* locus and might target other genes.^10^, ^16^, ^18^ These findings are in line with a previous study of our group suggesting the existence of two subtypes of B‐cell malignancies with *BCL3*‐translocations: the first subtype mostly diagnosed as CLL and characterized by few chromosomal changes, particular trisomy 12, and unmutated *IGHV* genes; and the second subtype including B‐cell malignancies of various histologic subtypes containing a relatively high number of chromosomal changes and mostly mutated *IGHV* genes.^4^ The predominance of cases with unmutated *IGHV* genes^9^, ^10^, ^27^ and high frequent trisomy 12 has meanwhile been corroborated by various studies in the CLL‐type of *BCL3*‐rearranged B‐cell malignancies,^9^, ^10^, ^17^ whereas investigations of recurrently mutated genes in several publications revealed a heterogeneous picture.^10^, ^11^, ^12^

Although these outlined studies have significantly advanced our understanding of *IGH*::*BCL3*‐positive B‐cell neoplasms, small sample sizes have often limited them. Therefore, we here aimed to genetically and epigenetically characterize a large cohort of B‐cell neoplasms with *IGH*::*BCL3*‐translocation from 84 patients, of which 80 were diagnosed with CLL. Besides, we moreover analyzed the DNA methylome of the cohort in comparison to CLL without *IGH*::*BCL3*‐translocation and investigated the clinical outcome associated with the translocation. Our findings provide further evidence that CLL with *IGH*::*BCL3*‐translocation constitutes a separate subtype within the CLL spectrum of mature leukemic B‐cell malignancies with distinct but homogeneous genetic, epigenetic, and clinical features.

## METHODS

### Patient and control samples

**Figure 1:**
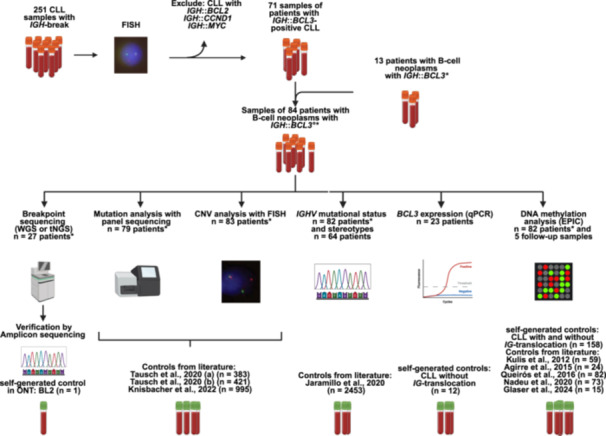
**Workflow of the selection of 84 patients with B‐cell neoplasms with *immunoglobulin heavy chain* (*IGH*)::*B‐cell lymphoma 3* (*BCL3*)‐translocation.** CLL, chronic lymphocytic leukemia; CNV, copy number variant; FISH, fluorescence in situ hybridization; ONT, Oxford Nanopore Technologies; qPCR, quantitative polymerase chain reaction; tNGS, targeted next‐generation sequencing; WGS, whole‐genome sequencing. °In 67/84 patients, an *IGH*::*BCL3*‐fusion was detected by FISH. In 17/84 patients, an *IGH* break and a *BCL3* break were detected by FISH. *Non‐CLL samples included.

We analyzed a total of 251 patient samples of leukemic mature B‐cell malignancy with evidence for a chromosomal translocation involving the *IGH* locus other than *IGH*::*BCL2*, *IGH*::*MYC*, or *IGH*::*CCND1* based on fluorescence in situ hybridization (FISH) analyses. Through testing at the Department of Internal Medicine 3 of the Ulm University Medical Center within this cohort, we identified 71 patients (collected between 1998 and 2020) diagnosed with CLL and confirmed *IGH::BCL3*‐translocation. In addition, 13 patients with B‐cell neoplasms subjected to diagnostic detection of an *IGH*::*BCL3*‐translocation at the Institute of Human Genetics at Christian‐Albrechts‐University Kiel were included with sample 11 already published in a previous paper of our group.^4^ Thus, the total cohort comprised samples of 84 patients with B‐cell neoplasms with *IGH*::*BCL3*‐translocation (see Figure 1). The initial diagnoses were CLL (*n* = 80), MZL (*n* = 2), mantle cell lymphoma (MCL; *n* = 1), or non‐Hodgkin lymphoma, not other specified (NHL, NOS; *n* = 1). Furthermore, from 4/80 patients with CLL, a total of five additional samples were studied at follow‐up for their DNA methylation. For comparison, 44 CLL samples without an *IG*‐translocation (32 for DNA methylation analyses and 12 for quantitative polymerase chain reaction [qPCR]), 4 CLL samples with *IGH*::*BCL2*‐translocation, 20 CLL samples with *IGH*::*MYC*‐translocation, and 102 CLL samples with *IGH* break and unknown partner from the Department of Internal Medicine 3 of the Ulm University Medical Center were investigated. Tumor DNA and RNA were extracted from peripheral blood or bone marrow samples (see Supporting Information S1: Tables 1 and 2). Control DNA for long‐read sequencing was extracted from a BL2 cell line (Burkitt lymphoma cell line lacking *IGH*::*BCL3*‐translocation).

For clinical comparison, an efficacy cohort consisting of 4237 previously untreated patients with CLL enrolled in the trials CLL4, CLL5, CLL8, CLL10, CLL11, CLL13, CLL14, CLL2‐GIVe, and CLL2‐BAG/BCG/BIO/BIG of the German CLL Study Group (GCLLSG) between 1994 and 2019 was studied. FISH results for the *IGH* break were available for 3832 patients of these cases (90.4%). For 28 patients with *IGH*::*BCL3*‐translocation enrolled in trials of the GCLLSG with first‐line therapy, a survival analysis was performed compared to patients without *IGH*::*BCL3*. Patient characteristics and outcome of seven of these patients were also included in a recently published ERIC study.^28^

### Fluorescence in situ hybridization and conventional cytogenetics

Cells extracted from peripheral blood or bone marrow were subjected to FISH analyses. For detection of breakpoints in the *IGH* and *BCL3* loci, a commercial *IGH* break‐apart (BA) probe (Vysis LSI IGH Dual Color, Break Apart Rearrangement Probe, Abbott, Chicago, IL, USA) as well as a non‐commercial *BCL3* BA probe^4^ were used. For the detection of the *IGH*::*BCL3* fusion, a non‐commercial *IGH*::*BCL3* dual‐color dual‐fusion FISH probe was applied to patients with sufficient material (67/84). In the remaining 17 patients, the *IGH*::*BCL3* fusion was inferred from BA patterns in both *IGH* and *BCL3* loci (of these, the presence of the fusion was verified by sequencing in samples of 5 patients). For the detection of chromosomal imbalances, 83 patient samples were studied on fixed cells extracted from peripheral blood or bone marrow using probes for deletion 11q, deletion 17p, deletion 13q, and trisomy 12. Preparation of FISH probes, hybridization, washing, counterstaining, evaluation, and documentation followed recently described protocols.^29^, ^30^, ^31^ The frequency of chromosomal imbalances was compared with the copy number variants (CNVs) data of the DNA methylation data and data of published CLL populations.^32^, ^33^, ^34^ The FISH assays are listed in Supporting Information S1: Table 3. Conventional cytogenetic analysis was performed according to the described protocols.^4^ A karyotype was defined as complex if ≥3 chromosomal aberrations in the same clone (numerical and/or structural; balanced aberrations were considered as a single event, unbalanced aberrations were considered as two events) were present in conventional cytogenetics (CC).^35^

### Targeted capture‐based and whole‐genome sequencing

To identify the translocation breakpoints, custom whole‐genome sequencing (WGS) of tumor samples of 17 patients at ATLAS Biolabs GmbH (Berlin, Germany) and Novogene (Martinsried, Germany), and in‐house targeted capture‐based sequencing of tumor samples of 10 patients were performed (for protocol details, bioinformatic analysis, and analysis of breakpoint junctions see Supporting Information S2: Methods). For breakpoint sequences, see Supporting Information S1: Table 4. The list of genes included in the targeted capture‐based assay is detailed in Supporting Information S1: Table 5. The positions for the class‐switch regions in the *IGH* locus were obtained from Hübschmann et al.^36^ In selected cases, long‐read sequencing using Oxford Nanopore Technologies (ONT, Oxford, UK) and Sanger Sequencing for verifying junctional segments were performed in addition. Moreover, qPCR was performed to study *BCL3* transcriptional expression in a subset of 23 cases with *IGH*::*BCL3*‐translocation. These methods are described in detail in Supporting Information S2: Methods and Supporting Information S1: Tables 6 and 7.

### CLL candidate gene, *IGHV* mutation, and stereotype subset analyses

To identify recurrently mutated candidate genes, we investigated the DNA of 79 patients with *IGH*::*BCL3*‐translocated B‐cell neoplasms using a custom Illumina AmpliSeq library covering 15 genes (see Supporting Information S2: Methods) either for the full genes or the most commonly affected exons.^33^ Bioinformatic evaluation was performed as described recently^33^ and detailed in the Supporting Information S2: Methods. The frequency of mutations was compared with publicly available data from CLL populations not stratified based on genomic characteristics.^32^, ^33^, ^34^ *IGHV* mutation determination and stereotype subset analyses were performed as described^37^, ^38^ and detailed in the Supporting Information S2: Methods, along with statistical analyses and visualization.

### DNA methylation analysis

DNA methylation of samples of 82 patients with *IGH*::*BCL3*‐translocation, 5 additional follow‐up samples from four of the 82 patients and 32 CLL samples without *IG*‐translocation, 4 CLL samples with *IGH*::*BCL2*‐translocation, 20 CLL samples with *IGH*::*MYC*‐translocation and 102 CLL samples with *IGH* break and unknown partner were examined using Infinium® MethylationEPIC BeadChip (EPIC) arrays following the manufacturer's recommendations (Illumina, San Diego, CA, USA). Publicly available data of the Infinium® Human Methylation 450 BeadChip (HM450K) array or EPIC arrays from 59 CLL samples,^39^ 155 MCL samples,^40^, ^41^ 59 follicular lymphoma (FL) samples, 8 nodal MZL (nMZL) samples,^42^ and 24 multiple myeloma (MM) samples^43^ were additionally used as controls. Normalization and analysis of DNA methylation data including the generation of CNV plots (segment median cutoff > 0.1 or <−0.1) were done as published recently.^42^ A total of 441,870 CpGs passed the quality assessment and entered the downstream analysis described in the Supporting Information S2: Methods.

### Development of a binary *BCL3* classifier using DNA methylation data

To develop a binary classifier capable of differentiating unmutated *IGH*::*BCL3*‐translocated CLL samples from the unmutated CLL samples with other *IG*‐translocation (see Patient and Control samples and additional publicly available data^39^), we performed a dimensional reduction over the 10,000 most variable CpGs from the DNA methylation data of the HM450K and EPIC array. The CpGs were clustered using k‐means, and selected CpGs were analyzed in further detail using different sampling techniques, model training, test, and evaluation. Finally, summary plots using Shapley Additive exPlanations (SHAP) values were generated to distinguish between unmutated CLL samples with *IGH*::*BCL3*‐translocation from other unmutated CLL samples with and without *IG*‐translocation (for details see Supporting Information S3: Figure 1 and Supporting Information S2: Methods).

## RESULTS

### Study cohort of B‐cell neoplasms with *IGH*::*BCL3*‐translocation

In the present study, we included samples from B‐cell neoplasms from 84 patients in which FISH using BA probes detected simultaneous breakpoints in the *IGH* and *BCL3* loci, suggestive of an *IGH*::*BCL3* fusion. An *IGH*::*BCL3*‐juxtaposition using a double‐color double‐fusion probe was verified by FISH in 67 patients with suitable material. In five additional cases, the fusion was proven by sequencing (see below), leaving 12 patients in which the *IGH*::*BCL3* fusion was inferred solely from BA patterns. As the overall cohort was initially screened for an *IGH* break, an inclusion criterion of the present study, the cohort was not systematically tested for *IGK* or *IGL* breaks or respective *IG light‐chain*::*BCL3* fusions. The total cohort consisted of 80 patients diagnosed with CLL, two diagnosed with MZL, one with a suspicious diagnosis of MCL, and one with NHL, NOS. A total of 60% (50/84) of the patients were males. Among the 68 patients with available data, the median age at diagnosis was 61 (range 27–83) years (see Supporting Information S1: Table 1).

### Identification of *IGH*::*BCL3*‐junctional sequences and genomic distribution of 19q13.32 breakpoints

**Figure 2:**
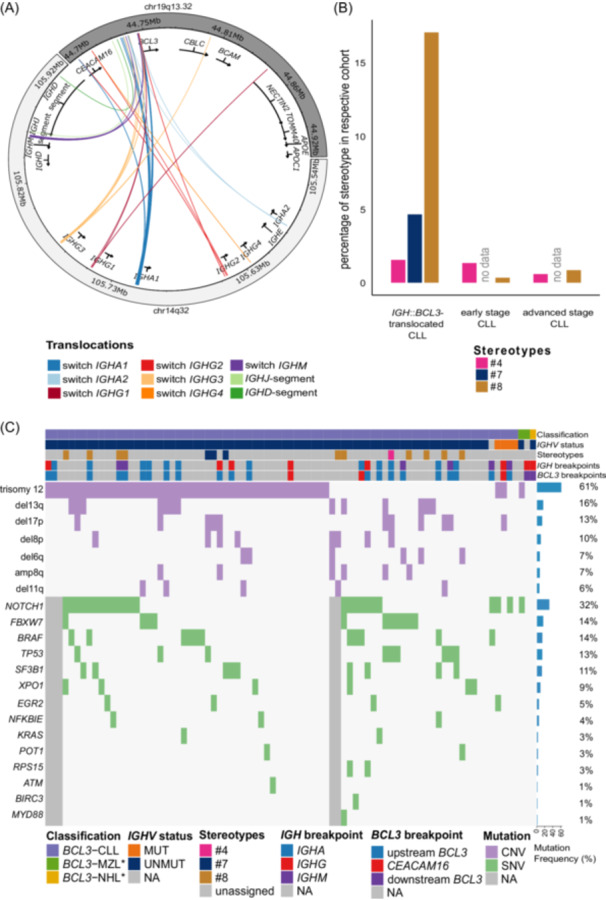
**Genetic characterization of *immunoglobulin heavy chain* (*IGH*)::*B‐cell lymphoma 3* (*BCL3*)‐translocated B‐cell neoplasms**. **(A)** Circleplot of the *IGH* loci in chromosome 14 (light gray) and the *BCL3* loci in chromosome 19 (dark gray, GRCh38). The breakpoints are colored according to their *IGH*‐location. The *IGHV*‐locus is not displayed because no breakpoints were found in there. **(B)** Barplot of the percentage of different stereotypes in *IGH*::*BCL3*‐translocated chronic lymphocytic leukemia (CLL) (*n* = 64, left panel) compared to early (*n* = 592; middle panel, Sonia Jaramillo et al.^38^) and advanced stage CLL (*n* = 1861; right panel, Sonia Jaramillo et al.^38^). **(C)** Oncoplot of recurrently mutated genes and copy number variants in CLL samples with *IGH*::*BCL3*‐translocation. Top annotations show classification, *IGHV* mutation status, stereotypes, *IGH* breakpoints, and *BCL3* breakpoints. The aberrations on the *y*‐axis are sorted according to their frequency. *Non‐CLL samples included.

To characterize the *IGH*::*BCL3*‐translocation junctions on both derivative chromosomes, der(14) and der(19), we subjected 27 patient samples (24 CLL, 1 MCL, 1 NHL, and 1 MZL) to targeted breakpoint or WGS. As per the sample, each derivative chromosome has a chromosome 14 and a chromosome 19 breakpoint; this results if both partner chromosomes are present in a total of theoretically 108 breakpoints. This considers that the rearrangements need not be balanced and that alterations (e.g., deletions, N‐nucleotides) might occur at the breakpoints. From the 108 theoretical breakpoints, we identified 106, i.e., in all cases subjected to sequencing, we verified the presence of FISH‐detected *IGH*::*BCL3* fusion. Notably, in one of the 27 samples (Case 69), we identified two breakpoints (hg38: chr19:44701841 and chr14:105711229) with additional long‐read sequencing, which were not detectable by short‐read targeted capture‐based sequencing. The two other breakpoints in case 69 (one breakpoint in chromosome 19 and one breakpoint in chromosome 14) were detected with both approaches and were identical. In chromosome 19q13, 49 of 53 (92%) breakpoints were located upstream (i.e., centromeric, 5′) of *BCL3*. The breakpoints were scattered over a region of more than 40 kb (hg38: chr19:44701327–44749070). The four most centromeric breakpoints (hg38: chr19:44701841–44701976) representing both derivative chromosomes 14 and 19 of two patients were located in intron 2 of the *CEACAM16* gene. Long‐read sequencing revealed an allele‐specific hypomethylated region at an enhancer site^44^ distal to the 19q13 breakpoint but upstream of *BCL3* in one of the latter cases. This could potentially associate with transcriptional deregulation of *BCL3* (see Supporting Information S3: Figure 2).

Breakpoints located upstream of *BCL3* were from cases diagnosed with CLL and one with a suspicious diagnosis of MCL (no *IGH*::*CCND1* fusion). Of the 53 breakpoints in 19q13, 4 (8%, two breakpoints per case) mapped downstream (3′, telomeric) of *BCL3* and were located in intron 9 of *CBLC* in a case diagnosed as NHL NOS, and 375 bp upstream of *NECTIN2*, in a case diagnosed as MZL (see Figure 2A).

### Expression of *BCL3* transcripts in CLL samples with *IGH*::*BCL3*‐translocation

**Figure 3:**
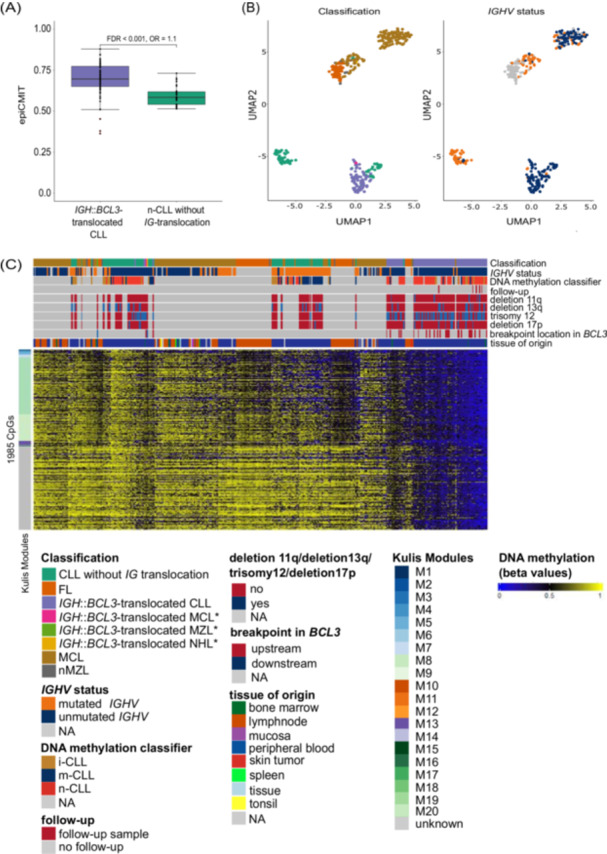
**Characterization of DNA methylation data from 82 *immunoglobulin heavy chain* (*IGH*)::*B‐cell lymphoma 3* (*BCL3*)‐translocated B‐cell neoplasms and five follow‐up samples**. **(A)** Boxplot with *IGH*::*BCL3*‐translocated chronic lymphocytic leukemia (CLL) samples (*n* = 78 without follow‐up samples) and naïve‐like CLL (n‐CLL) samples (*n* = 32) without *immunoglobulin* (*IG*)‐translocation as control on the *x*‐axis and the epiCMIT on the *y*‐axis showing the significantly higher epiCMIT of CLL samples with *IGH*::*BCL3*‐translocation compared to n‐CLL samples without *IG*‐translocation. FDR, false discovery rate. **(B)** UMAP analyses of the 1000 most variable CpGs from CLL samples with *IGH*::*BCL3*‐translocation (*n* = 83, including five follow‐up samples), mantle cell lymphoma (MCL) with *IGH*::*BCL3*‐translocation (*n* = 1), marginal zone lymphoma (MZL) with *IGH*::*BCL3*‐translocation (*n* = 2), non‐Hodgkin lymphoma (NHL) with *IGH*::*BCL3*‐translocation (*n* = 1), CLL without *IG*‐translocation (*n* = 91), follicular lymphomas (FLs, *n* = 59), MCL (*n* = 155), and nodal marginal zone lymphomas (nMZLs, *n* = 8). The left UMAP panel represents the classification of the different samples (green: CLL without *IG*‐translocation; orange: FL; purple: *IGH*::*BCL3‐*translocated CLL; pink: *IGH*::*BCL3*‐translocated MCL; light green: *IGH*::*BCL3*‐translocated MZL; yellow: *IGH*::*BCL3*‐translocated NHL; brown: MCL; and gray: nMZL). The right UMAP represents the *IGHV* mutational status of the different samples (orange: mutated *IGHV*; dark blue: unmutated *IGHV*; and gray: data not available). **(C)** Heatmap of a supervised analysis of unmutated *IGH*::*BCL3*‐translocated CLL samples (including five follow‐up samples) and unmutated CLL samples without *IG*‐translocation (FDR < 0.01; |Δ*β* | > 0.3) showing the 1985 differentially methylated CpGs. CpGs are listed per row, and samples are listed per column. The top annotation displays classification, *IGHV* status, DNA methylation classifier, follow‐up sample, possible deletion 11q, deletion 13q, trisomy 12, and deletion 17p, breakpoint location in the *BCL3* locus, and the tissue of origin. The left annotation represents the Kulis Modules. i‐CLL, intermediate CLL; m‐CLL, memory‐like CLL; n‐CLL, naïve‐like CLL. *Non‐CLL samples included. Low DNA methylation values are colored in blue and high DNA methylation values in yellow.

To investigate the effect of the *BCL3*‐translocation on its expression, we measured *BCL3* transcriptional levels by qPCR in upstream *IGH*::*BCL3*‐translocated CLL samples of 23 patients and 12 CLL samples without *IG*‐translocation. We observed a significantly higher expression of *BCL3* in the *IGH*::*BCL3*‐translocated CLL samples compared to the CLL samples without *IG*‐translocation (P = 0.0375, see Supporting Information S3: Figure 3).

### Mapping of *IGH* breakpoints and characterization of the mechanism underlying the illegitimate recombination

To elucidate the mechanism underlying the illegitimate *IGH* recombination leading to *IGH*::*BCL3* fusion, we mapped the breakpoints in 14q32 to the functional segments of the *IGH* locus. A total of 53 breakpoints were located in one of the class‐switch recombination (CSR) regions in the *IGH* locus. The breakpoints were located in *IGHA1* (*n* = 19/53 breakpoints in 11 patient samples), *IGHM* (*n* = 10/53 breakpoints in 5 patient samples), *IGHG3* (*n* = 9/53 breakpoints in 5 patient samples), *IGHG2* (*n* = 5/53 breakpoints in 3 patient samples), *IGHG1* (*n* = 4/53 breakpoints in 3 patient samples), *IGHA2* (*n* = 3/53 breakpoints in 2 patient samples) and *IGHG4* (*n* = 1/53 breakpoint in 1 patient sample) switch regions. The one remaining patient sample showed breakpoints within the *IGHJ6* and *IGHD1–7* genes (see Figure 2A). Due to the presence of recombination signal sequences (RSS) sites (RSS12 and RSS23: each located 12 bp away from the breakpoint in *IGHD1‐7*) and N‐nucleotides (11 and 6 bp) between the breakpoints, we assume aberrant VDJ recombination mediated the *IGH*::*BCL3* junctions in this case. We conclude that the vast majority of illegitimate *IGH*::*BCL3* rearrangements occur due to aberrant class‐switching with a high frequency of defective *IGHA1* CSR.

### *IGHV* mutation status and stereotypes in cases with *IGH*::*BCL3*‐translocation

Next, we investigated the *IGHV* mutation status in 82/84 patients of our cohort. In 95% (78/82) of the evaluable patients, *IGHV* was unmutated, and in 5% (4/82), *IGHV* was mutated (homology 87% to 95%). In addition, we performed a stereotype subset analysis of 64 patients with CLL of our cohort. In 15/64 patient samples, a stereotype subset was assigned, which was subset #8 in 11/64 (17%), #7 in 3/64 (5%), and #4 in 1/64 (2%) patient samples (see Figure 2B). In comparison to early and advanced stage CLL populations,^38^ we detected a significant enrichment of subset #8 in the *IGH*::*BCL3* translocated CLL samples (for early stage CLL: false discovery rate [FDR] < 0.001; OR: 61; for advanced stage CLL: FDR < 0.001; OR: 24). We conclude, that *IGH*::*BCL3* translocated CLL samples carry predominately unmutated *IGHV* genes and show a strong bias towards subset #8.

### Copy number variants and mutational landscape of cases with *IGH*::*BCL3*‐translocation

**Figure 4:**
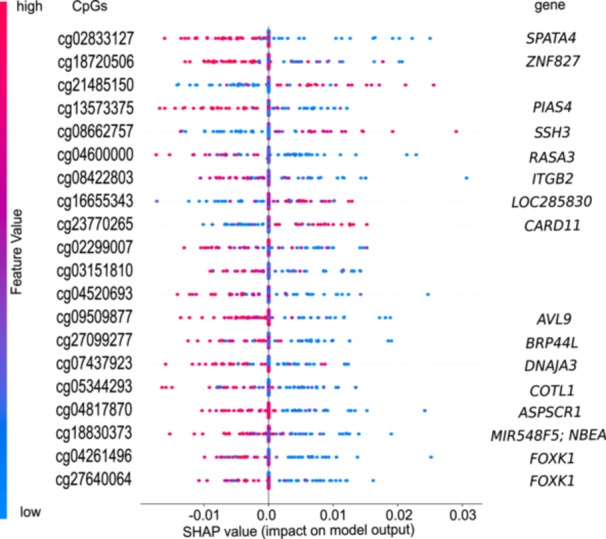
**Shapley Additive exPlanations (SHAP) summary plot for the *B‐cell lymphoma 3* (*BCL3*) classifier.** It displays SHAP values for each data point in the dataset. Each row corresponds to a feature (CpG). Colors indicate feature values—red for high and blue for low. Points further to the right have a positive impact in predicting *IGH*::*BCL3* on the model's output, while those to the left have a negative impact. The gene annotation according to the CpGs is displayed on the right side.

Furthermore, we called CNVs in the B‐cell neoplasms with *IGH*::*BCL3*‐translocation using interphase FISH and DNA methylation array data (for comparison, see Supporting Information S2: Results). In the 83 patients with available FISH data, trisomy 12 was detected in 61% of cases (51/83), deletion 17p in 13% of cases (11/83), and deletion 11q in 6% (5/83) (see Figure 2C, Supporting Information S3: Figure 4A, and Supporting Information S1: Table 8). For the analysis of deletion 13q, we relied on the FISH data, due to the small size of the deletion, and detected a deletion 13q in 16% of cases (13/83). In contrast, data for deletion 8p, deletion 6q and gain of 8q were only obtained from the DNA methylation arrays and showed deletion 8p in 10% (8/82), including three cases with loss of *TNFRSF10B*, deletion 6q in 7% (6/82) and gain of 8q in 7% (6/82) of cases with two of the latter cases also carrying a concurrent deletion of 8p (see Figure 2C and Supporting Information S1: Table 9). In comparison to the published CLL population,^32^, ^33^, ^34^ trisomy 12 (FDR < 0.001; OR: 9), deletion 8p (FDR < 0.005; OR: 3), deletion 6q (FDR < 0.05; OR: 3), and gain of 8q (FDR < 0.05; OR: 3) were significantly enriched, whereas deletion 13q (FDR < 0.001; OR: 0.2) was significantly underrepresented in B‐cell neoplasms with *IGH*::*BCL3*‐translocation (see Supporting Information S3: Figure 4B).

In addition to CNVs, we investigated karyotype complexity in cases with data from CC. We identified at least three chromosomal aberrations in 40% (4/10) of patients with one patient (CLL_4) diagnosed as MZL and three as CLL.

Next, we investigated the presence of mutations in genes frequently targeted by small variants in CLL in *IGH*::*BCL3*‐positive samples of 79 patients from our series using panel sequencing with a median depth of 3018x. There was good agreement between the deep targeted sequencing and the WGS with a notable absence of *BCL3* coding mutations or signs of ongoing somatic hypermutation in *BCL3* in the latter. For the comparison of CLL candidate gene mutations from targeted sequencing and WGS data, see Supporting Information S2: Results. We detected somatic variants in *NOTCH1* in 32% (25/79), *FBXW7* and *BRAF* in 14% (11/79), *TP53* in 13% (10/79), *SF3B1* in 11% (9/79), *XPO1* in 9% (7/79), *EGR2* in 5% (4/79), and *NFKBIE* in 4% (3/79), *KRAS, POT1*, and *RPS15* in 3% (2/79) in each, as well as *ATM*, *BIRC3*, and *MYD88* in 1% (1/79) of *IGH*::*BCL3*‐positive samples. In comparison to the published CLL population,^32^, ^33^, ^34^ we observed significant enrichment for variants in *NOTCH1* (FDR < 0.001; OR: 3), *BRAF* (FDR < 0.001; OR: 4), and *FBXW7* (FDR < 0.001; OR: 5) and significant depletion of variants in *ATM* (FDR < 0.05; OR: 0.1) (see Figure 2C, Supporting Information S3: Figure 4B, and Supporting Information S1: Table 10). Three of the four non‐CLL cases (two MZL cases and one NHL case) were included in the mutational analysis, with one MZL case carrying a *NOTCH1* variant (NM_017617:c.*378A>G).

Combining CNV and mutation analysis, we identified 15 patients with *IGH*::*BCL3*‐positive B‐cell neoplasms affected by both a *NOTCH1* variant and a trisomy 12. However, formal analysis showed that the likelihood of co‐occurrence did not exceed chance (P = 1, OR: 1). In contrast, we identified significant co‐occurrence of *TP53* variant and deletion 17p (P < 0.001; OR: 30).

We conclude that compared to common CLL, the *IGH*::*BCL3* translocated B‐cell neoplasms are enriched for trisomy 12, show a reduced frequency of deletion 13q, and display a spectrum of somatic variants particularly affecting *NOTCH1*, *FBXW7*, and *BRAF*.

### DNA methylation profile of B‐cell neoplasms with *IGH*::*BCL3*‐translocation

**Figure 5:**
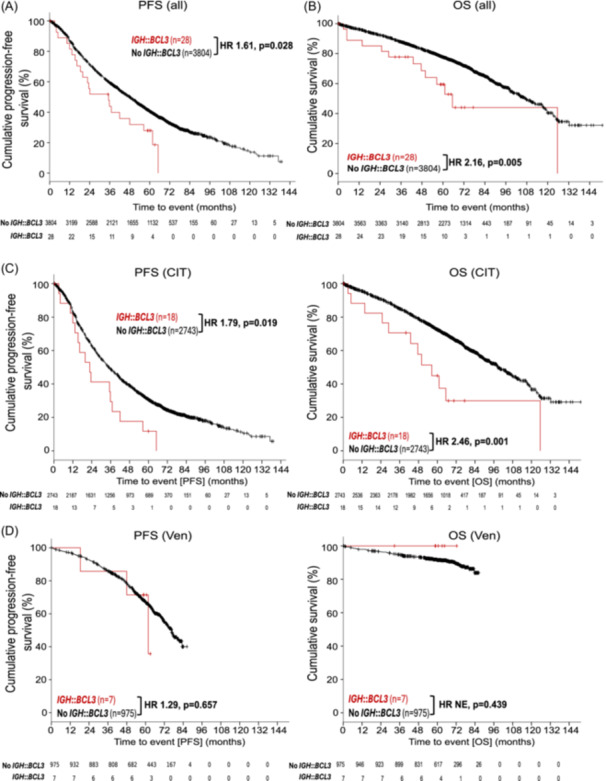
**Kaplan–Meier estimates of *immunoglobulin heavy chain* (*IGH*)*::B‐cell lymphoma 3* (*BCL3*)‐translocated chronic lymphocytic leukemia (CLL) samples treated in German CLL Study Group (GCLLSG) trials.** Survival plot for progression‐free survival (PFS) **(A)** and overall survival (OS) **(B)** of the full cohort (trials: CLL4, CLL5, CLL8, CLL10, CLL11, CLL13, CLL14, CLL2‐GIVe, and CLL2‐BAG/BCG/BIO/BIG) and for subgroups according to treatment with chemo(immune)therapy (CIT) **(C)** and venetoclax‐based treatments (Ven) **(D)**. 95% confidence interval (CI) for A: 1.048–2.474. 95% CI for B: 1.249–3.735. 95% CI for C (PFS): 1.093–2.924. 95% CI for C (OS): 1.393–4.356. 95% CI for D (PFS): 0.415–4.032. 95% CI for D (OS): not evaluable (NE). HR, hazard ratio. *IGH*::*BCL3*‐translocated CLL samples are depicted in red, and CLL samples without *IGH*::*BCL3*‐translocation are depicted in black.

Finally, we analyzed the DNA methylation profiles for 82 patients with B‐cell neoplasms with *IGH*::*BCL3*‐translocation including the 25 samples with breakpoints upstream of *BCL3* and two samples with breakpoints downstream of *BCL3*. DNA methylation in five follow‐up samples of *IGH*::*BCL3*‐positive CLL from four patients showed that the correlation between initial and follow‐up sample (follow‐ups between 1 and 10 years after initial sample) was significantly higher (*t*_Welch_(4.02) = 7.70, P = 1.50e^−03^, *g*_Hedges_ = 2.54, CI_95%_[0.74, 4.34]) than between non‐related *IGH*::*BCL3*‐positive CLL samples (see Supporting Information S3: Figure 5) indicating the DNA methylation to stay stable during the course of the disease even as the underlying genetic landscape undergoes changes including the gain of one *SF3B1* variant (NM_012433: exon14: c.A1996C, p.K666Q) and one *TP53* variant in follow‐up CLL_R5 of case CLL18, the gain of one *NFKBIE* variant (*NFKBIE*:NM_004 556: exon1: c.342_345del, p.Y115Sfs*13) in follow‐up CLL_R1 of case CLL84, and the loss of deletion 11q with concurrent gain of deletion13q in follow‐up CLL_R3 of case CLL81 (see Supporting Information S1: Table 11).

Applying the CLL epitype classifier,^45^ 62% (51/82) of initial patient samples were defined as naïve‐like CLL (n‐CLL), including one case of MZL, 10% (8/82) as intermediate CLL (i‐CLL), and 9% (7/82) as memory‐like CLL (m‐CLL, see Supporting Information S1: Table 1). The remaining 16/82 patients were called “unclassified” due to their tumor cell content being below 60%. Notably, the latter cases with tumor cell content below 60% according to this classifier also included the two samples with breakpoints downstream of *BCL3* and the one case suspicious of MCL (see Supporting Information S1: Table 1). Thus, only taking the samples with sufficient tumor cell content into account, 51/66 (77%) were classified as n‐CLL, 8/66 (12%) as i‐CLL, and 7/66 (11%) as m‐CLL. Considering cases with respective data, all cases assigned to the n‐CLL and all cases assigned to the i‐CLL groups exhibited an unmutated *IGHV* status. Among the m‐CLL samples, three out of seven displayed a mutated *IGHV* status, while the remaining four out of seven exhibited an unmutated *IGHV* status. Four out of five follow‐up samples were classified as n‐CLL, consistent with their initial classifications. We compared the findings in the *IGH*::*BCL3*‐positive B‐cell neoplasms (without follow‐ups) with published data from CLL populations^46^ and detected an enrichment of n‐CLL (FDR < 0.01; OR: 3) and a depletion of m‐CLL (FDR: < 0.001; OR: 0.1) in our cohort.

To investigate the proliferative history of *IGH*::*BCL3*‐translocated CLL samples (excluding follow‐ups), we applied the epiCMIT tool^45^ and compared them to n‐CLL samples without *IG*‐translocation and all CLL samples without *IG*‐translocation. We detected a significantly higher epiCMIT‐measured proliferative history of B‐cell neoplasms with *IGH*::*BCL3*‐translocation in comparison to the n‐CLL (FDR < 0.001, OR: 1.1, see Figure 3A and Supporting Information S3: Figure 6C).

To investigate whether the epigenetic profile of *IGH*::*BCL3*‐translocated B‐cell neoplasms (including follow‐ups) is distinct from other B‐cell neoplasms, we compared the DNA methylation pattern with publicly available and in‐house data of CLL samples without *IG*‐translocations, FL, nMZL, and MCL samples.^39^, ^40^, ^41^, ^42^ An UMAP analysis using the 1000 most variable CpGs showed that the CLL segregated according to *IGHV* mutation status. *IGH*::*BCL3*‐translocated CLL samples (80 CLL samples with unmutated *IGHV* status including follow‐up samples) defined an individually segregating group in the UMAP analysis (see Figure 3B).

Based on the findings from the UMAP analysis, we next performed a supervised analysis between unmutated *IGH*::*BCL3*‐translocated CLL samples (including follow‐ups) and unmutated CLL samples without *IG*‐translocation (FDR < 0.01; |Δ*β* | > 0.3). This analysis revealed 1985 differentially methylated CpGs with *IGH*::*BCL3*‐translocated CLL samples showing lower DNA methylation at 1960 CpGs assigned to 971 genes and higher methylation at 25 CpGs assigned to 9 genes (see Figure 3C and Supporting Information S1: Table 12). We compared the identified CpGs with the modules defined by Kulis et al.^47^ describing sets of CpG sites that share similar DNA methylation dynamics during B‐cell differentiation. Enrichment of hypomethylated CpGs in *IGH*::*BCL3*‐positive CLL samples was observed in Kulis Modules 8 (929/1960 CpGs), 9 (390/1960 CpGs), and 13 (64/1960 CpGs) (FDR < 0.001; OR > 1) (see Supporting Information S3: Figure 6A). Additionally, the 1960 hypomethylated CpGs were predominantly located in heterochromatic regions (61%, 1194/1960) (see Supporting Information S3: Figure 6B). Notably, Kulis Modules 8 and 9 are characterized by heterochromatic demethylation during late B‐cell differentiation. Given that heterochromatic demethylation is a hallmark epigenetic change in MM,^48^ we compared the DNA methylomes of 24 previously published MM cases^43^ with our set of 1985 differentially methylated CpGs. We observed similar DNA methylation patterns between MM cases and *IGH*::*BCL3*‐translocated CLL samples for the 1063 Kulis‐associated CpGs. Specifically, 80% (855/1063) of these CpGs displayed low DNA methylation levels in MM, while 20% (208/1063) exhibited higher DNA methylation levels (see Supporting Information S3: Figure 7). Therefore, the *IGH*::*BCL3*‐translocated CLL samples show a DNA methylation pattern distinct from other CLL samples, which is characterized by features of unmutated CLL with moreover signs of increased proliferative history and additional loss of DNA methylation in heterochromatic regions.

### Development of a binary *BCL3* classifier using DNA methylation data

Given the strong DNA methylation differences between *IGH*::*BCL3*‐translocated and other CLL samples, we finally aimed at developing a binary classifier to differentiate unmutated *IGH*::*BCL3*‐translocated CLL samples (including follow‐ups) from unmutated CLL samples with other *IG*‐translocations and those without *IG*‐translocations. The model exhibited outstanding performance with an AUC‐ROC (area under the receiver operating characteristic curve) of 0.99 and an AUC‐PRC (precision‐recall curve) of 0.98, reflecting its excellent discriminative ability and precision‐recall balance (see Supporting Information S3: Figure 8A,B). It achieved an average precision of 98% for both classes, with recall rates of 96% for *IGH*::*BCL3* and 94% for other CLL. The F1 scores were 94% for *IGH*::*BCL3*‐translocated and 96% for other CLL, underscoring the model's robustness in accurately identifying *IGH*::*BCL3* instances. We applied SHAP for interpretability to the classifier process and identified 20 CpG sites (see Figure 4 and Supporting Information S1: Table 13) as the most influential features for the model's decision‐making process regarding *IGH*::*BCL3* classification. Interestingly, high CpG DNA methylation values were indicative of *IGH*::*BCL3* in only four instances. Vice versa, 16 CpG sites were associated with low DNA methylation values in *IGH*::*BCL3* translocated CLL samples (see Supporting Information S3: Figure 8C).

### *IGH*::*BCL3* is an adverse prognostic factor in CLL

Besides the genetic and epigenetic differences of *IGH*::*BCL3*‐translocated CLL as compared to other CLL samples, we finally investigated the clinical impact of the *IGH*::*BCL3*‐translocation in patients with CLL. To this end, we focused on the 28/80 patients diagnosed with *IGH::BCL3*‐translocation positive CLL in this study, which were also enrolled in trials of the GCLLSG. Among a total number of 3832 previously untreated patients with valid FISH result for *IGH* break analysis from the GCLLSG trials, the median follow‐up was 71.2 months. During this period, there were 2396 events for progression‐free survival (PFS) and 1098 for overall survival (OS). Patients with *IGH*::*BCL3* translocated CLL were either treated with chemo(immune)therapy (*n* = 18) or time‐limited venetoclax‐based treatments (*n* = 7), with three patients receiving other therapy. Patients with CLL harboring the *IGH*::*BCL3*‐translocation had a significantly shorter PFS (HR 1.61, CI95% 1.05‐2.47, P = 0.03) and OS (HR 2.16, CI95% 1.25‐3.74, P < 0.01) compared to those without (see Figure 5). This adverse prognostic impact was observed in the subgroup of patients being treated with chemoimmunotherapy, whereas it was absent in those being treated with venetoclax‐based regimens. Notably, the estimated 5‐year OS rate was reduced in patients with *IGH*::*BCL3*‐positive CLL treated with chemo(immuno)therapy (44.9% vs. 72.9%), while all seven patients with *IGH*::*BCL3*‐positive CLL treated with venetoclax remained alive corresponding to an estimated 5‐year OS rate of 100% versus 91.5% for patients without the translocation. Considering *IGHV* mutation status, OS of patients with *IGH*::*BCL3*‐translocated CLL was significantly shorter compared to patients with CLL without translocation and unmutated *IGHV* (P = 0.05), whereas PFS did not differ significantly (see Supporting Information S3: Figure 9).

## DISCUSSION

In this study, we analyzed B‐cell neoplasms with *IGH*::*BCL3*‐translocation from 84 patients, revealing distinct genetic and epigenetic features. Key findings in these neoplasms include breakpoints upstream of *BCL3*, driven by aberrant CSR commonly involving *IGHA1*, unmutated *IGHV* status with occurrence of CLL stereotype subset #8, and specific alterations including trisomy 12 and *NOTCH1*. *IGH*::*BCL3*‐translocated CLL samples showed higher *BCL3* expression and a unique DNA methylation profile. The latter showed features of naïve B‐cell‐like CLL but in addition strong loss of methylation at heterochromatic sites and signs of increased proliferative history. Moreover, DNA methylation at 20 CpG sites was able to robustly distinguish *IGH*::*BCL3*‐positive CLL from other CLL subtypes. Finally, analysis of the subset of patients included in trials of the GCLLSG revealed that *IGH*::*BCL3*‐translocated CLL was associated with shorter PFS and OS with chemoimmunotherapy in comparison to CLL without this translocation but not with venetoclax.

Our genetic findings revealed breakpoints primarily upstream of *BCL3* in CLL cases and downstream in MZL and NHL cases, consistent with a recent study.^10^ *IGH*::*BCL3*‐translocated CLL samples with upstream *BCL3* breakpoints showed deregulated *BCL3* expression. The *IGH* breakpoints are strongly associated with unmutated *IGHV*, indicating that these CLL do not undergo somatic hypermutation in the germinal center. In line, we did not detect significant traces of somatic hypermutation at the *BCL3* locus. Breakpoints predominantly arise from aberrant CSR.^10^, ^16^, ^18^ While evidence for a T‐cell dependent class‐switch outside the germinal center has been shown in murine models,^49^ this has not been clearly demonstrated in humans. Alternatively, a T‐cell‐independent CSR may also occur outside the germinal center.^50^, ^51^ This suggests that CLL samples with *IGH*::*BCL3*‐translocations originate from non‐germinal center B cells. Our analysis revealed 44% of breakpoints in *IGHA* class‐switch loci, particularly *IGHA1*, possibly related to IgA's unique role in mucosal immunity.^52^ However, the stereotype subset #8 enriched in our cohort (11/15 cases) as well as in earlier reports on *IGH*::*BCL3*‐positive CLL,^53^ is usually associated with surface IgG expression.^54^, ^55^ This seems to conflict with the high frequency of *IGHA*‐associated translocations in our cohort, which are usually related to *IGHA* germline transcription. The number of cases with breakpoint assignments in the subset #8 was too low for any conclusions, but remarkably, the breakpoints affected the *IGHA* switch region only in 1/3 cases with the subset #8 genotype. Co‐occurrence of stereotype subset #8 with trisomy 12^38^ was detected in 4/11 cases in our cohort in line with published findings.^4^, ^9^, ^10^, ^17^, ^27^ Additionally, *TP63* overexpression has been previously also described together with subset #8 in *IGH*::*BCL3*‐translocated CLL in the literature,^10^ which might underscore a unique combination of genetic and transcriptional events in these CLL. In line, and given the involvement of *BCL3* in NF‐κB signaling, our study and others^10^, ^11^, ^12^ revealed a remarkable enrichment of mutations in NF‐κB signaling genes (*NOTCH1*, *BRAF*, and *FBXW7*).

Epigenetic analyses clearly distinguished the *IGH*::*BCL3‐*translocated CLL samples from both MCL and nMZL samples in the UMAP analysis but also with the 20 CpGs detected by the *BCL3* classifier (see Figure 3B,C and Supporting Information S3: Figure 8) and highlighted an unmutated naïve‐like B‐cell signature with signs of increased proliferative history compared to CLL samples without *IG*‐translocation.^39^ The DNA methylation patterns show an additional loss of DNA methylation in heterochromatic regions that somewhat resembles the epigenetic patterns in plasma cells or plasma cell neoplasias.^48^ In this context, high CD38 expression in nine *IGH*::*BCL3*‐translocated CLL cases is notable, which is a feature common to MM^56^ but also unmutated CLL.^57^

The distinct DNA methylation profile of *IGH*::*BCL3‐*translocated CLL samples allowed us to develop a binary classifier that distinguishes unmutated CLL samples with *IGH*::*BCL3*‐translocation from other unmutated CLL samples. Despite using only 20 CpG sites, the classifier is highly robust with F1 scores of 94% for *IGH*::*BCL3*‐translocated and 96% for other CLL. Though not designed as a biologically interpreted selection of features, it is notable that the classifier included one CpG located in a superenhancer of *CARD11* (cg23770265).^58^ Given the role of *CARD11* in B‐cell receptor‐induced NF‐κB activation^59^ and the function of *BCL3* as transcriptional coactivator of the NF‐κB signaling pathway,^6^ it is intriguing to speculate that the DNA methylation properties of *IGH*::*BCL3* translocated CLL samples to some extent reflect the pathway deregulation. Additionally, the binary methylation classifier presented herein not only captures the distinct epigenetic signature of *IGH*::*BCL3*‐translocated CLL samples but also carries practical potential to distinguish these cases from other unmutated CLL samples particularly if no data on *IGH* translocation partners from conventional cytogenetic, FISH, or molecular studies are obtained.

Clinically, B‐cell neoplasms with *IGH*::*BCL3*‐translocation are diagnosed at a younger median age (61 years in our cohort, 52–69 years in literature^4^, ^9^, ^10^, ^16^, ^28^) compared to typical CLL (median age at diagnosis: 65–70 years^60^, ^61^). In the literature, the clinical outcome of patients with *IGH*::*BCL3*‐rearranged CLL has been reported with conflicting results, with some studies suggesting adverse features^5^, ^9^ while others^10^, ^53^ finding no significant difference compared to patients with CLL with unmutated *IGHV*. During the revision of this paper, a study with 101 patients with *IGH*::*BCL3*‐translocated CLL detected a shorter time to treatment and OS compared to patients with untranslocated CLL.^28^ This observation was confirmed by us in an analysis of a large cohort examining the impact of *IGH*::*BCL3* in patients enrolled in clinical trials. In patients with CLL carrying the translocation, treatment with chemoimmunotherapy was associated with significantly shorter PFS and OS, whereas outcomes with the *BCL2* inhibitor venetoclax were unaffected. Similar results were reported by Visentin et al. (2025), who observed a non‐significant trend to prolonged time‐to‐next‐treatment (TTNT) with venetoclax in *IGH*::*BCL3*‐translocated CLL.^28^ This difference may be explained by the upregulation of *BCL3*, which acts as a coactivator of NF‐κB and induces anti‐apoptotic gene expression independent of *BCL2*.^62^ The observed associations between *IGH*::*BCL3*‐translocation and clinical outcomes are based on univariable analyses; confounding effects from other prognostic factors cannot be excluded, and multivariable modeling was not feasible due to the limited sample/event number.

In summary, our analysis of *IGH*::*BCL3*‐translocated B‐cell neoplasms of 84 patients suggests that the CLL samples carrying this aberration form a distinct subtype within the CLL spectrum. *IGH*::*BCL3*‐translocated CLL samples show genetic and epigenetic features of non‐germinal center B‐cells with naïve B‐cell‐like DNA methylation and *IGHV* mutation pattern, increased proliferation history and additional loss of DNA methylation in heterochromatic regions, and are enriched in stereotype subset #8. Breakpoints mostly occur due to faulty switch recombination mostly with *IGHA*. Trisomy 12 and mutations in NF‐κB signaling genes are recurrent, and the disease presents at a younger age than common CLL with shorter PFS and OS with chemoimmunotherapy treatment. Based on these homogeneous characteristics, which are nevertheless distinct from the main subgroups of CLL, we propose that *IGH*::*BCL3‐*translocated CLL constitutes a separate entity within the spectrum of mature B‐cell leukemias/CLL. The differential diagnosis of these leukemias can be reliably achieved by a variety of methods already in used routine diagnostics like CC, FISH, or breakpoint determination by sequencing‐based strategies as well as by DNA methylation analysis as presented herein.

## AUTHOR CONTRIBUTIONS

**Cosima Drewes**: Writing—original draft; visualization; validation; methodology; software; formal analysis. **Cristina López**: Writing—review and editing; supervision; methodology; project administration. **Juan Emilio Martinez‐Manjón**: Software. **Nnamdi Okeke**: Methodology. **Billy Jebaraj**: Methodology. **Susanne Bens**: Supervision. **Björn Brändl**: Methodology. **Martin J. S. Dyer**: Writing—review and editing; resources. **Barbara Eichhorst**: Resources. **Anja Fischer**: Writing—review and editing; software; formal analysis. **Kirsten Fischer**: Resources. **Sandra Robrecht**: Formal analysis. **Michael Hallek**: Resources. **Selina Glaser**: Software; formal analysis; writing—review and editing. **Sina Hillebrecht**: Methodology. **Sandrine Jayne**: Resources. **Helene Kretzmer**: Software; formal analysis. **Anja Mottok**: Project administration. **Franz‐Josef Müller**: Software; methodology. **Christof Schneider**: Resources. **Ole Ammerpohl**: Software. **Coral del Val**: Software; formal analysis. **Stephan Stilgenbauer**: Resources; supervision. **Eugen Tausch**: Supervision; resources; methodology; writing—review and editing. **Reiner Siebert**: Writing—review and editing; funding acquisition; supervision.

## CONFLICT OF INTEREST STATEMENT

The authors declare no conflicts of interest.

## ETHICS STATEMENT

The study has been approved by the Ethics Committee of the Medical Faculty of Ulm University (No. 464/19) and was conducted in accordance with the guidelines of the Institutional Review Boards of Christian‐Albrecht‐University Kiel (D447/10, Amendment 2.11.2015).

## FUNDING

This work has been supported by the Deutsche Forschungsgemeinschaft SFB1074 B10N and Spanish Ministry of Science and Technology project RTI2018‐098983‐B‐100 and “Ethical, Responsible, and General‐Purpose Artificial Intelligence: Applications in Risk Scenarios (IAFER)” Exp.: TSI‐100927‐2023‐1. Open Access funding enabled and organized by Projekt DEAL.

## Supporting information

Supporting Information.

Supporting Information.

Supporting Information.

## Data Availability

The data that support the findings of this study are openly available in GEO at https://www.ncbi.nlm.nih.gov/geo/, reference number GSE295456. DNA methylome data produced in this study are available at GEO under accession number GSE295456. Whole‐genome sequencing data and targeted breakpoint NGS will be available under restricted access.
