# Supplementary material for: Chronic lymphocytic leukemia with IGH::BCL3‐translocation is characterized by a homogeneous and distinct genetic and epigenetic landscape
Source: Hemasphere. 2026 Apr 29;10(5):e70354. doi: 10.1002/hem3.70354 (PMC13126245; doi:10.1002/hem3.70354)
Supplement: Supplementary file 2 — Supporting Information. [file HEM3-10-e70354-s001.docx]

**Supplementary Information**

**Methods**

**Targeted capture-based and Whole Genome Sequencing (WGS)**

To identify the translocation breakpoints, custom WGS of tumor samples of 17 patients was performed with the TruSeq PCR-free Library Preparation (2x150bp or 350bp) on a NovaSeq (Illumina, San Diego, CA, USA) by a respective service provider (Novogene, Martinsried, Germany or ATLAS Biolabs GmbH, Berlin, Germany). In-house targeted capture-based sequencing of tumor samples of ten patients was performed with the SureSelectXT HS Target Enrichment System (2x150bp) and sequenced with NextSeq 550 (Illumina, San Diego, CA, USA). The coverage (meandepth) of each sample was calculated using samtools (version 1.19.2) (1). The mean coverage for each sample was calculated only including the meandepth of chromosomes 1 to 22 for each sample for the WGS data. For the targeted capture-based approach the mean coverage for each sample was calculated only including the meandepth of the *IGH*-locus (hg38: chr14: 105479370-106470468) and *BCL3*-locus (hg38: chr19:40278931-40320151). The mean and median of the mean coverages per sample were calculated using R software (version 4.3.1). The median of the mean coverages per sample in the whole genome sequences was 37x (range: 29x to 48x) and for the targeted capture-based approach 145x (range: 41x to 258x). The processed fastq files were mapped to the human reference genome (GRCh38) using BWA-MEM (version 2.2.1) (2). The BAM files were generated, sorted and indexed using samtools (version 1.19.2) (1). Quality control was performed with FastQC (version 0.12.1) (3). Immunoglobulin translocations were called by Igcaller (version 1.3) (4) and visually inspected using the Integrative Genomic Viewer (IGV, version 2.17.0) (5).

**Analysis and verification of breakpoint junctions at the translocated *IGH* gene locus**

The extracted *IGH*::*BCL3* junctional sequences were inspected with Human BLAT Search from UCSC Genome Browser (https://genome.ucsc.edu/cgi-bin/hgBlat, viewed at 09.12.24) identifying N-nucleotides at the breakpoint junctions and the RSS database CNR-ITB was used for the analysis (https://www.itb.cnr.it/rss/index.html, viewed at 22.06.24) to detect the presence of cryptic recombination signal sequences (RSS) within 1kb up- and downstream of the breakpoints in chromosome 14 and 19. The system determines if genetic sites are functional based on RIC scores, following Cowell's research (6–8). For RSS-23 sites, a score of -58.45 or higher means the site is functional (passes). For RSS-12 sites, a score of -38.81 or higher indicates functionality (passes). Scores below these thresholds mean the sites are non-functional (fail) (6–8).

**Long-read sequencing using Oxford Nanopore Technologies (ONT)**

Genomic DNA of one *IGH*::*BCL3* positive CLL sample was extracted using the DNA easy blood + tissue Kit (Quiagen, Hilden, Germany), according to the manufacturer´s manual. The DNA was dissolved overnight at 4°C and quantified by using the Nanodrop (Thermo Fisher Scientific, Waltham, MA, USA). For the patient DNA, 5 µg of the extracted DNA were used in total as input for nanopore library preparation with the ligation sequencing DNA V12 kit (SQK-LSK112 from ONT, UK) according to the manufacturer´s protocol (ONT, Oxford, UK). The library was split up in five and was loaded on five Gridion R10.4 flow cells (FLO-MIN112 from ONT, Oxford, UK) and sequenced with a Gridion device for up to 72 h. Sequencing was performed using the Minknow software (version 22.10.7). Fast5 files were converted to pod5 files using pod5 convert (Pod5 version: 0.3.21). Basecalling was performed with dorado basecaller (version 4.1.0; https://github.com/nanoporetech/dorado, viewed at 28.11.24) with “sup”: parameter ‘—trim’ and model ‘dna_r10.4.1_e8.2_400bps_sup@v4.1.0‘ and mod-base model ‘dna_r10.4.1_e8.2_400bps_sup@v4.1.0_5mCG_5hmCG@v2’. Alignment was performed using minimap2 "--secondary=no -2 -a -y” (version 2.26-r1175) (9).

Genomic DNA of a *IGH*::*BCL3* negative cell line control (BL2) was extracted using the DNA easy blood + tissue Kit (Quiagen, Hilden, Germany), according to the manufacturer´s manual. 12 µg of the extracted DNA was used in total as input for nanopore library preparation with the ligation sequencing DNA kit (SQK-LSK109-XL from ONT, Oxford, UK) according to the manufacturer´s protocol. The library was split up in three and was loaded on a Promethion flow cell (FLO-PRO002 from ONT, Oxford, UK) and sequenced with a Promethion device for up to 72 h. Throughout the sequencing process, the flow cells were reloaded one time after a nuclease flush (EXP-WSH004 from ONT, Oxford, UK) with the same library according to the manufacturer´s instructions, to enhance sequencing yield. Sequencing was performed using the Minknow software (Minknow CORE 4.3.4, Bream 6.2.5, Guppy 5.0.11). Basecalling was performed with guppy basecaller (version 5.0.11) with “hac”: parameter. Alignment was performed using minimap2 "--secondary=no -2 -a -y” (version 2.26-r1175) (9).

The aligned BAM files were subjected to downstream analyses. The Epi2Me human variation workflow (version 1.9.0, available at <https://github.com/epi2me-labs/wf-human-variation>, viewed at 28.11.24) was executed to generate DNA methylation data, structural variant data, and phased aligned BAM files (based on the GRCh38 reference genome) for the whole genome. A minimum coverage threshold of 1 was applied. Additionally, the BAM files underwent manual inspection using the Integrative Genomic Viewer (IGV, version 2.17.4).

**Breakpoint verification by PCR and Sanger Sequencing**

The breakpoints of five samples with *IGH*::*BCL3* translocations and one sample with *IGH*::*NECTIN2* translocation, as detected through WGS or targeted capture-based sequencing, underwent verification via Sanger sequencing. This involved primers positioned in proximity to the breakpoints. The PCR primers and conditions utilized for Sanger sequencing are provided in Supplementary Table 5. Sequencing of the PCR products was carried out employing the Big Dye Terminator v3.1 Cycle Sequencing Kit (Applied Biosystems, Waltham, MA, USA), followed by analysis of the sequences utilizing an 3130xl Genetic Analyzer (Applied Biosystems, Waltham, MA, USA).

**CLL candidate gene mutation analysis**

To identify recurrently mutated candidate genes, we investigated DNA of *IGH*::*BCL3*-translocated B-cell neoplasms of 79 patients using a custom Illumina AmpliSeq library covering *NOTCH1* (NM_017617)*, SF3B1* (NM_012433)*, ATM* (NM_000051)*, TP53* (NM_001126115)*, RPS15* (NM_001018.5)*, BIRC3* (NM_001165)*, MYD88* (NM_001172568)*, FBXW7* (NM_001013415)*, POT1* (NM_015450)*, XPO1* (NM_001378470)*, NFKBIE* (NM_004556)*, EGR2* (NM_000399)*, BRAF* (NM_001378470)*, NRAS* (NM_002524) and *KRAS* (NM_001369786) either for the full gene or the most commonly affected exons (10). Adjacent 5bp in the intron were included to cover splice site mutations. Sequencing was performed on an Illumina MiSeq™ in 48 sample batches with the 600-cycle MiSeq Reagent Kit v3. For alignment, variant calling and annotation we used a custom bioinformatics pipeline including BWA-MEM (version 2.2.1) (2) and samtools for alignment (version 1.19.2) (1), and Varscan2 for variant calling and annotation (11) and Scandel for deletion calling. Current databases (COSMIC, 1000G, dbSNP150, ClinVar, Seshat) were taken into consideration to evaluate and report variants above a threshold of 5 % mean variant allele fraction (VAF) as pathogenic/non pathogenic. Only variants classified as pathogenic or likely pathogenic were considered as mutations, while single nucleotide polymorphisms (SNPs) with frequency > 1 % in healthy population, variants classified as benign or likely benign or variants of unknown significance were not reported.

**Reverse transcription and quantitative polymerase chain reaction (qPCR)**

Samples of 23 patients with *IGH*::*BCL3*-translocated CLL and 12 CLL without *IG* -translocation (as controls) were used for qPCR-based measurement of *BCL3* transcriptional levels. cDNA synthesis was performed using GoScript™ Reverse Transcription System (Promega, Madison, WI, USA). In brief, RNA was isolated from patient PBMCs using Qiagen RNeasy mini kit. 400 ng of RNA was reverse transcribed using random hexamers according to the manufacturer’s instructions. The synthesized cDNA mix was diluted 1:10 v/v. For qPCR, the iTaq Universal SYBR Green Supermix (Bio-Rad, Hercules, CA, USA) was used. PCR reactions were set up on MicroAmp optical 384 well plates (Applied Biosystems, Waltham, MA, USA) in a total volume of 10 µl per well. Primer sequences for housekeeping genes *Actin B* and *RPL19* and the targets gene *BCL3* are mentioned in Supplementary Table 6. Final concentration of each primer used for qPCR was 500 nM. qPCR was performed in a QuantStudio 5 qPCR-system (Applied Biosystems, Waltham, MA, USA). PCR amplification was performed for 40 cycles using denaturation at 95°C for 15 seconds followed by a one step annealing and elongation at 60°C for 1 minute as recommended in the product’s specifications.

***IGHV* mutation status and stereotype subset analysis**

*IGHV* mutation status was assessed from 82 patient samples by amplifying the predominant VDJ rearrangement of the heavy chain using multiplex PCR according to literature (12). The sequence of the tumor sample was compared to the germline sequence with highest homology. The *IGHV* mutation status was calculated as the ratio of the number of matching nucleotides in the *HV* gene and the total number of nucleotides in the *IGHV* germline gene. An unmutated *IGHV* mutation status was defined by an identity to the germline of 98 % or higher. *IGHV* clonal sequence analysis was implemented as a standard procedure beginning in 2021. Consequently, we were able to examine 64 samples for their stereotype classification. Stereotype analyses were performed by amplification of the *IGHV*-*IGHD*-*IGHJ* gene rearrangements and subsequent Sanger sequencing to determine to the common sequence. By uploading the sequence to ARResT (https://bat.infspire.org/arrest/assignsubsets/) an assignment to one of nineteen major CLL stereotyped subsets according to relative scores and confidence assignment is performed (13). The CLL with *IGH*::*BCL3*-translocation were compared with publicly available data for stereotypes #1, #2, #4 and #8 of early and advanced stage CLL (13).

**DNA methylation analysis**

CLL tumors were classified into naive-like CLL (n-CLL), intermediate CLL (i-CLL) and memory-like CLL (m-CLL) using the CLL epitype classifier (version 2.1) (14). The 1000 most variable CpGs were extracted and UMAP analysis was performed. Differentially methylated CpGs of supervised analyses were extracted using limma package (version 3.58.1). CpGs were annotated using modules described by Kulis et al. (15) and chromatin states (16). The proliferative history was calculated using the epigenetically-determined cumulative mitoses (epiCMIT) tool (version 2.0) (14).

**Development of a binary *BCL3* classifier using DNA methylation data**

In order to develop a binary classifier capable of differentiating unmutated *IGH*::*BCL3*-translocated CLL from the unmutated CLL with other *IG*-translocation (*IGH*::*BCL2*-translocation, *IGH*::*MYC*-translocation, CLL with *IGH*-break and unknown partner from the Department of Internal Medicine 3 of the Ulm University Medical Center) and without *IG*-translocation (from Department of Internal Medicine 3 of the Ulm University Medical Center and additional publicly available data (17)) we performed a dimensionality reduction technique over the 10000 most variable CpGs from the DNA methylation data of the HM450K and EPIC array. The CpGs were clustered using k-means with k = 1000 based on their values across all samples. We selected clusters that contained between 1 and 4 CpGs for in-depth analysis. These specific clusters enclosed a total of 1221 CpGs, which represented the highest variability within the sample. These CpGs were subsequently utilized for the development of the *BCL3* classifier. To ensure a balanced representation in both training and test sets, we divided the 1221 CpGs using a 70/30 split. The classifier was developed with Python 3.11 and Spyder 5.4.3. The models chosen for our study included the K-Nearest Neighbors (KNN), Support Vector Machine (SVM), Naive Bayes (NB), RandomForestClassifier (18), XGBClassifier (19), and MLPClassifier(18). Since the dataset was significantly imbalanced, only 78 unmutated *IGH*::*BCL3*-translocated CLL (five samples were follow-up samples of four patients) versus 166 unmutated CLL with and without *IG*-translocation (102 unmutated CLL with *IGH*-break and unknown partner, 4 unmutated *IGH*::*BCL2*-translocated CLL, 20 unmutated *IGH*::*MYC*-translocated CLL and 40 unmutated CLL without *IG*-translocation,) were necessary for the sampling techniques to ensure that the model is not biased towards the majority class and can accurately predict minority class instances. We tested oversampling techniques (SMOTE, Random Over Sampling (ROS), ADASYN, Borderline SMOTE, k-means SMOTE, and SVM SMOTE), subsampling techniques (Cluster Centroids, Condensed Nearest Neighbour, Edited Nearest Neighbors, Repeated Edited Nearest Neighbors All k-NN, Instance Hardness Threshold, Near Miss, Neighbourhood Cleaning Rule, One Side Selection, Tomek Links, and Random Under Sampling) and mixed techniques (SMOTEEN and SMOTE Tomek).

We employed a 5-fold cross-validation strategy, where the data was split into five subsets. The model was trained five times, each time using 4 parts to train and 1 part to validate, rotating the subset used for validation. This helps to ensure that the model generalizes well and is not overfitted to a single dataset. Along with this strategy, we tested all defined hyperparameters and selected the best combination for each model based on its performance in the cross-validation using GridSearchCV. Once the best hyperparameters were found, we retrained each model using the entire training dataset to ensure the best possible performance. Model evaluation included standard measures such as precision, recall, F1 score, area under the receiver operating characteristic curve (AUC-ROC), and area under the precision-recall curve (AUC-PRC).

To provide explainability to the model we used SHAP values (Shapley Additive exPlanations) (20) which quantify the impact of individual features (CpGs) on the model’s output, enabling both global and local interpretations of feature importance. We generated summary plots, both global and local, to improve model explainability for the *BCL3* classifier. These plots prioritize CpGs based on their importance in distinguishing between unmutated CLL with *IGH*::*BCL3* translocation from other unmutated CLL with and without *IG*-translocation (see Supplementary Figure 1).

**Statistical analyses and Visualization**

Statistical analyses of genomic and epigenomic data were performed using R software (version 4.3.1). For comparing independent groups with continuous variables, the Wilcoxon rank sum test was employed. In the case of categorical variables, Fisher's exact test was utilized for small sample sizes (< 5), while the Chi-Square test was applied for larger sample sizes (≥ 5) to determine odds ratios and p-values. To account for multiple comparisons, p-values were adjusted using the Benjamini-Hochberg method. Statistical significance was established when the adjusted p-value (FDR) was below 0.05. Cohen’s Kappa-test was determined with irr package in R (https://rdocumentation.org/packages/irr/versions/0.84.1, viewed at 20.02.25). The correlation of DNA methylation data between initial and follow-up samples versus non-related *IGH*::*BCL3* CLL was performed with ggstatsplot package in R (https://github.com/IndrajeetPatil/ggstatsplot, version 0.13.0, viewed at 31.03.25). Statistical analyses of the efficacy cohort were performed using SPSS (version 31.0.0.0) by applying Kaplan-Meier methodology, log-rank tests and Cox proportional hazards regression modelling. Figure 1 was created with Biorender.

**Results**

**Comparison of copy number aberrations from DNA methylation data and interphase FISH data**

We determined copy number variants (CNVs) in the B-cell neoplasms with *IGH*::*BCL3*-translocation using interphase FISH and DNA methylation array data. In the 81 samples where data was available from both approaches there was an agreement of the results regarding presence or absence of 100 % (κ = 1, p < 0.001) for deletion 11q and deletion 17p and an agreement of 98 % for trisomy 12 (κ = 0.976, p < 0.001). One case was borderline negative for trisomy 12 in the DNA methylation array approach but positive in FISH approach (FISH: 14 % of cells showed trisomy 12). Therefore, we used the FISH data for the analysis of trisomy 12 and detected trisomy 12 in 61 % of cases (51/83). Both approaches detected deletion 17p in 14 % of cases (11/81) and deletion 11q in 6 % (5/81) (see Figure 1C or Supplementary Figure 4A). The agreement between FISH and DNA methylation data for deletion 13q was lower (κ = 0.627, p < 0.001) with seven cases only positive for the FISH approach but not for the DNA methylation array. Because of the small size of the deleted region in 13q this is frequently not captured by the array approach due to the lower resolution. Therefore, we relied on the FISH data for detection of 13q deletion, which was detected in 16 % of cases (13/83). In contrast, data for deletion 6q was only obtained by the DNA methylation arrays and showed deletion 6q in 7 % of cases (6/82) (see Supplementary Table 7).

**Comparison of** **CLL candidate gene mutation analysis from** **panel sequencing and WGS data**

We compared the data of the targeted sequencing with the WGS data of 24 matching cases and detected 82% (14/17) of variants in WGS. One variant in *SF3B1* (NM_012433:exon14:c.1998C>G) was only detected in 3/38 reads in WGS and one variant in *TP53* (NM_001126115:exon3:c.298T>A) was detected in 3/20 reads in WGS. Therefore, both variants were only called as variant in the targeted sequencing approach and not in WGS. The variant NM_001126115:exon2:c.246_247del in *TP53* was not called in WGS but in targeted sequencing.

**References:**

1. Danecek P, Bonfield JK, Liddle J, Marshall J, Ohan V, Pollard MO, et al. Twelve years of SAMtools and BCFtools. GigaScience. 2021 Feb 1;10(2):giab008.

2. Vasimuddin Md, Misra S, Li H, Aluru S. Efficient Architecture-Aware Acceleration of BWA-MEM for Multicore Systems. In: 2019 IEEE International Parallel and Distributed Processing Symposium (IPDPS). 2019. p. 314–24.

3. Andrews S. FastQC: a quality control tool for high throughput sequence data [Internet]. 2010 [cited 2024 Feb 21]. Available from: https://www.bioinformatics.babraham.ac.uk/projects/fastqc/

4. Nadeu F, Mas-de-les-Valls R, Navarro A, Royo R, Martín S, Villamor N, et al. IgCaller for reconstructing immunoglobulin gene rearrangements and oncogenic translocations from whole-genome sequencing in lymphoid neoplasms. Nature communications. 2020;11(1):3390.

5. Robinson JT, Thorvaldsdottir H, Turner D, Mesirov JP. igv.js: an embeddable JavaScript implementation of the Integrative Genomics Viewer (IGV). Bioinformatics. 2023 Jan 1;39(1):btac830.

6. Lee AI, Fugmann SD, Cowell LG, Ptaszek LM, Kelsoe G, Schatz DG. A functional analysis of the spacer of V(D)J recombination signal sequences. PLoS Biol. 2003 Oct;1(1):E1.

7. Cowell LG, Davila M, Kepler TB, Kelsoe G. Identification and utilization of arbitrary correlations in models of recombination signal sequences. Genome Biol. 2002;3(12):RESEARCH0072.

8. Cowell LG, Davila M, Ramsden D, Kelsoe G. Computational tools for understanding sequence variability in recombination signals. Immunol Rev. 2004 Aug;200:57–69.

9. Li H. Minimap2: pairwise alignment for nucleotide sequences. Bioinformatics. 2018 Sep 15;34(18):3094–100.

10. Tausch E, Schneider C, Robrecht S, Zhang C, Dolnik A, Bloehdorn J, et al. Prognostic and predictive impact of genetic markers in patients with CLL treated with obinutuzumab and venetoclax. Blood. 2020 Jun 25;135(26):2402–12.

11. Koboldt DC, Zhang Q, Larson DE, Shen D, McLellan MD, Lin L, et al. VarScan 2: somatic mutation and copy number alteration discovery in cancer by exome sequencing. Genome Res. 2012 Mar;22(3):568–76.

12. Kröber A, Seiler T, Benner A, Bullinger L, Brückle E, Lichter P, et al. V(H) mutation status, CD38 expression level, genomic aberrations, and survival in chronic lymphocytic leukemia. Blood. 2002 Aug 15;100(4):1410–6.

13. Jaramillo S, Agathangelidis A, Schneider C, Bahlo J, Robrecht S, Tausch E, et al. Prognostic impact of prevalent chronic lymphocytic leukemia stereotyped subsets: analysis within prospective clinical trials of the German CLL Study Group (GCLLSG). haematol. 2020 Nov 1;105(11):2598–607.

14. Duran-Ferrer M, Clot G, Nadeu F, Beekman R, Baumann T, Nordlund J, et al. The proliferative history shapes the DNA methylome of B-cell tumors and predicts clinical outcome. Nature cancer. 2020;1(11):1066–81.

15. Kulis M, Merkel A, Heath S, Queirós AC, Schuyler RP, Castellano G, et al. Whole-genome fingerprint of the DNA methylome during human B cell differentiation. Nature genetics. 2015;47(7):746–56.

16. Bal E, Kumar R, Hadigol M, Holmes AB, Hilton LK, Loh JW, et al. Super-enhancer hypermutation alters oncogene expression in B cell lymphoma. Nature. 2022 Jul;607(7920):808–15.

17. Kulis M, Heath S, Bibikova M, Queirós AC, Navarro A, Clot G, et al. Epigenomic analysis detects widespread gene-body DNA hypomethylation in chronic lymphocytic leukemia. Nature genetics. 2012;44(11):1236–42.

18. Pedregosa F, Varoquaux G, Gramfort A, Michel V, Thirion B, Grisel O, et al. Scikit-learn: Machine Learning in Python. Journal of Machine Learning Research. 2012 Jan 2;12.

19. Chen T, Guestrin C. XGBoost: A scalable tree boosting system. Proceedings of the ACM SIGKDD International Conference on Knowledge Discovery and Data Mining. 2016;13-17-Augu:785–94.

20. Lundberg SM, Lee SI. A unified approach to interpreting model predictions. Advances in Neural Information Processing Systems. 2017;2017-Decem(Section 2):4766–75.
