# Supplementary material for: Chronic lymphocytic leukemia with IGH::BCL3‐translocation is characterized by a homogeneous and distinct genetic and epigenetic landscape
Source: Hemasphere. 2026 Apr 29;10(5):e70354. doi: 10.1002/hem3.70354 (PMC13126245; doi:10.1002/hem3.70354)
Supplement: Supplementary file 3 — Supporting Information. [file HEM3-10-e70354-s002.pdf]

Supplementary Figure 1

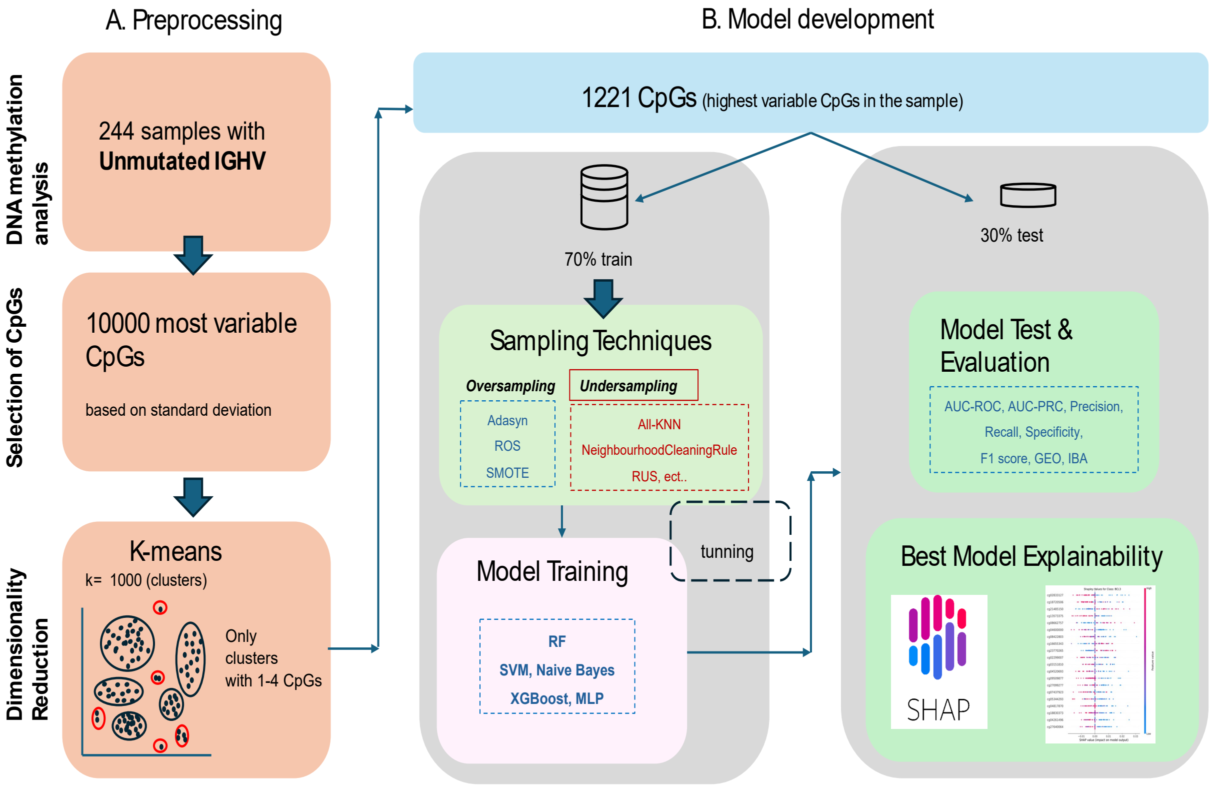

Supplementary Figure 1: Workflow of the *BCL3* classifier showing preprocessing and model development of the classifier. ROS = Random Over Sampling, KNN = K-Nearest Neighbours, AUC-ROC = area under the receiver operating characteristic curve, AUC-PRC = area under the precision-recall curve, SHAP = Shapley Additive exPlanations. The binary classifier was validated exclusively on HM450K and EPIC version 1 arrays, and it is likely not compatible with EPIC version 2.

Supplementary Figure 2

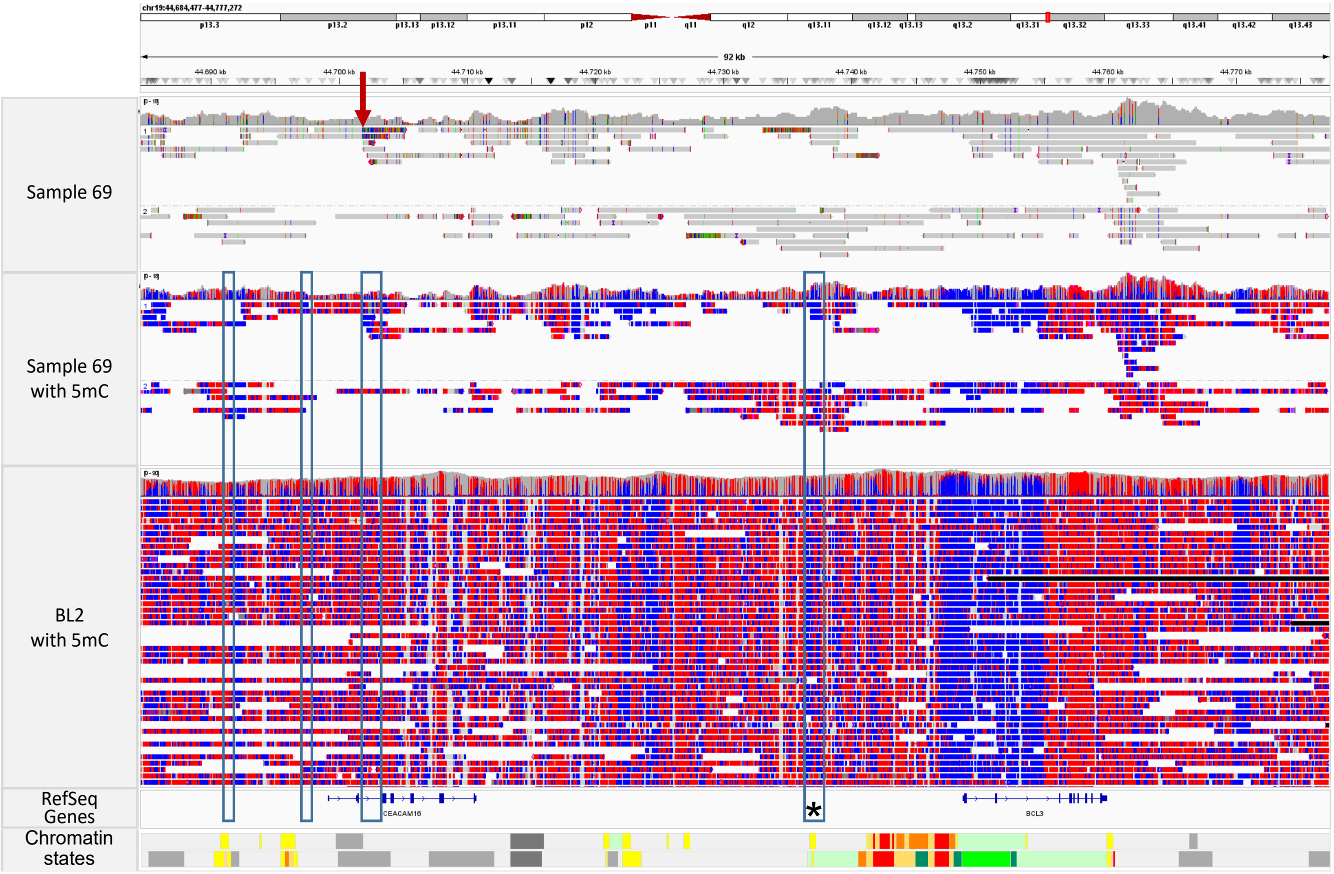

Supplementary Figure 2: Long-read sequencing data of sample 69 including DNA methylation data. IGV-view of patient sample 69 in chromosome 19 (hg38: chr19:44684477-44777272) with DNA methylation values (DNA-hypomethylation in blue and DNA-hypermethylation in red) and BL2 as control cell line performed by long-read sequencing. The red arrow represents the location of the two breakpoints in chromosome 19. The blue boxes mark the hypomethylated regions in patient sample 69 in comparison to BL2 (hg38: chr19:44691401-44692800, chr19:44697208-44697583, chr19:44701796-44703634 and chr19:44736209-44737541). * marks the enhancer site with allele-specific hypomethylation in sample 69. Chromatin states (yellow: weak enhancer, orange: strong enhancer, red: promotor, light green: weak transcription, dark green: strong transcription) in unmutated CLL are displayed according to Beekman et al., 2018.

Supplementary Figure 3

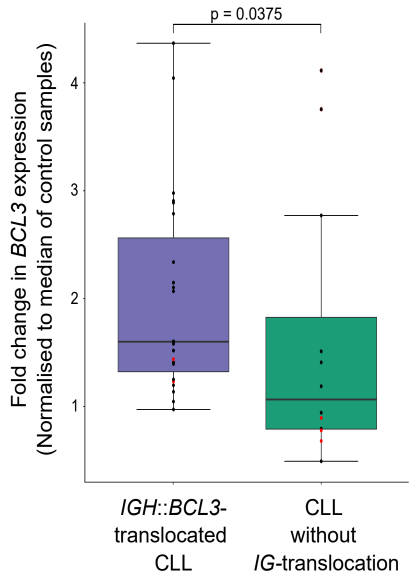

Supplementary Figure 3: *BCL3* expression in *IGH*::*BCL3*-translocated CLL samples. Boxplot with *IGH*::*BCL3*-translocated CLL samples (n = 23) and CLL samples without *IG*-translocation (n = 12) as control on x-axis and log2 fold change on the y-axis showing *BCL3* expression. Red color indicates samples with mutated *IGHV*.

Supplementary Figure 4

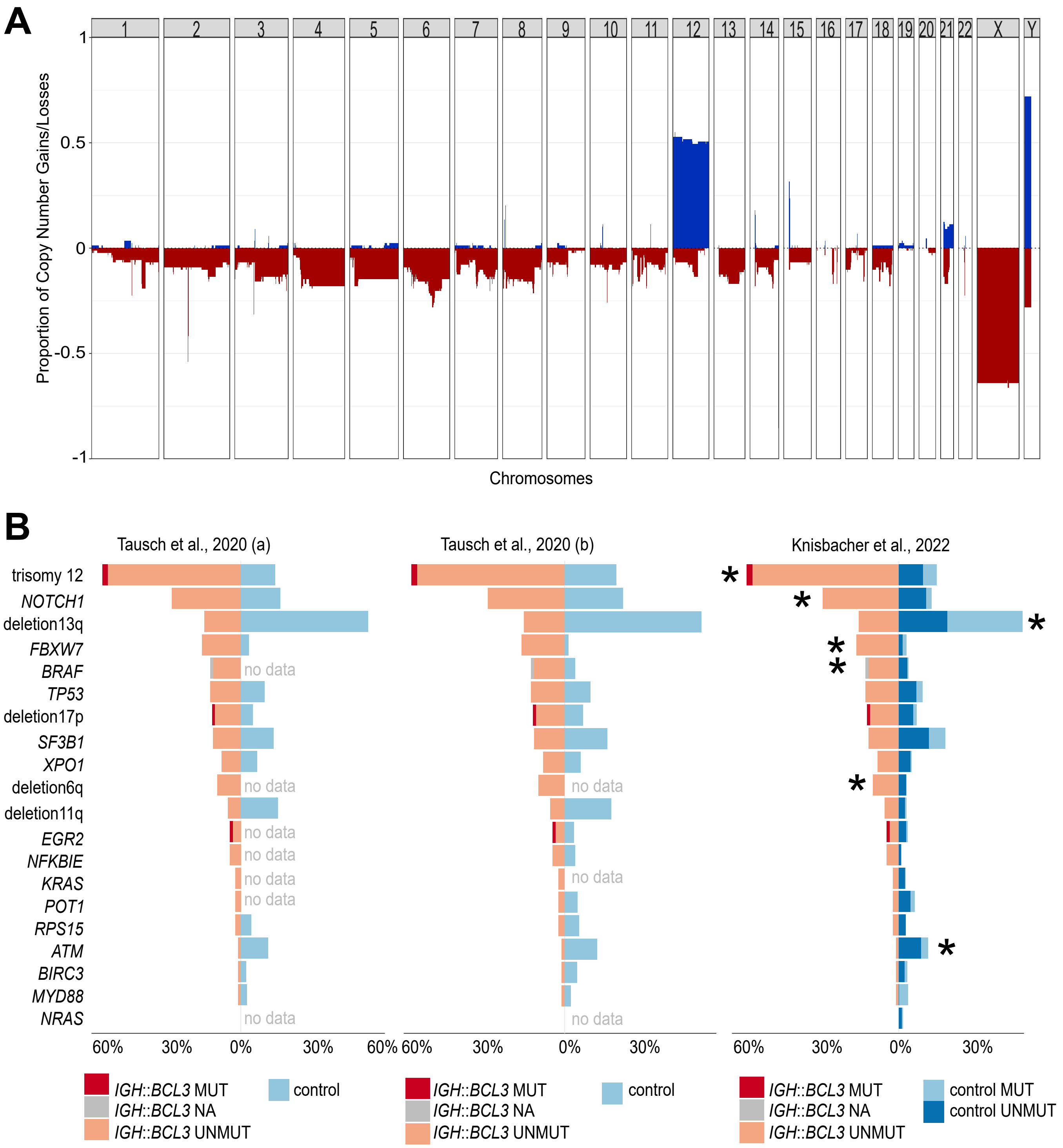

Supplementary Figure 4: Genetic aberrations in *IGH*::*BCL3*-translocated B-cell neoplasms*. A: Copy number variant (CNV)-overlayplot from DNA methylation data of 82 B-cell neoplasms* with *IGH*::*BCL3*-translocation and additional five follow-ups. The x-axis represents the different chromosomes and the y-axis represents the proportion of gains (in blue) and losses (in red). **B:** Barplots of percentage of different recurrently mutated genes and copy number variations in B-cell neoplasms* with *IGH*::*BCL3*-translocation (in red, n = 79) and data from three different CLL populations (in blue). * = FDR < 0.01, MUT = mutated *IGHV*, UNMUT = unmutated *IGHV*. * = non-CLL samples included

Supplementary Figure 5

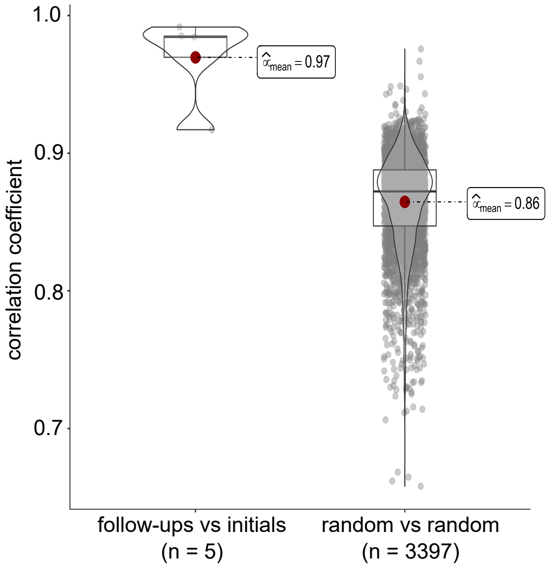

Supplementary Figure 5: Correlation coefficient of the DNA methylation data. Violinplot of the correlation coefficients of pairs of follow-up samples versus (vs) the initial samples in comparison to other not-related samples.

Supplementary Figure 6

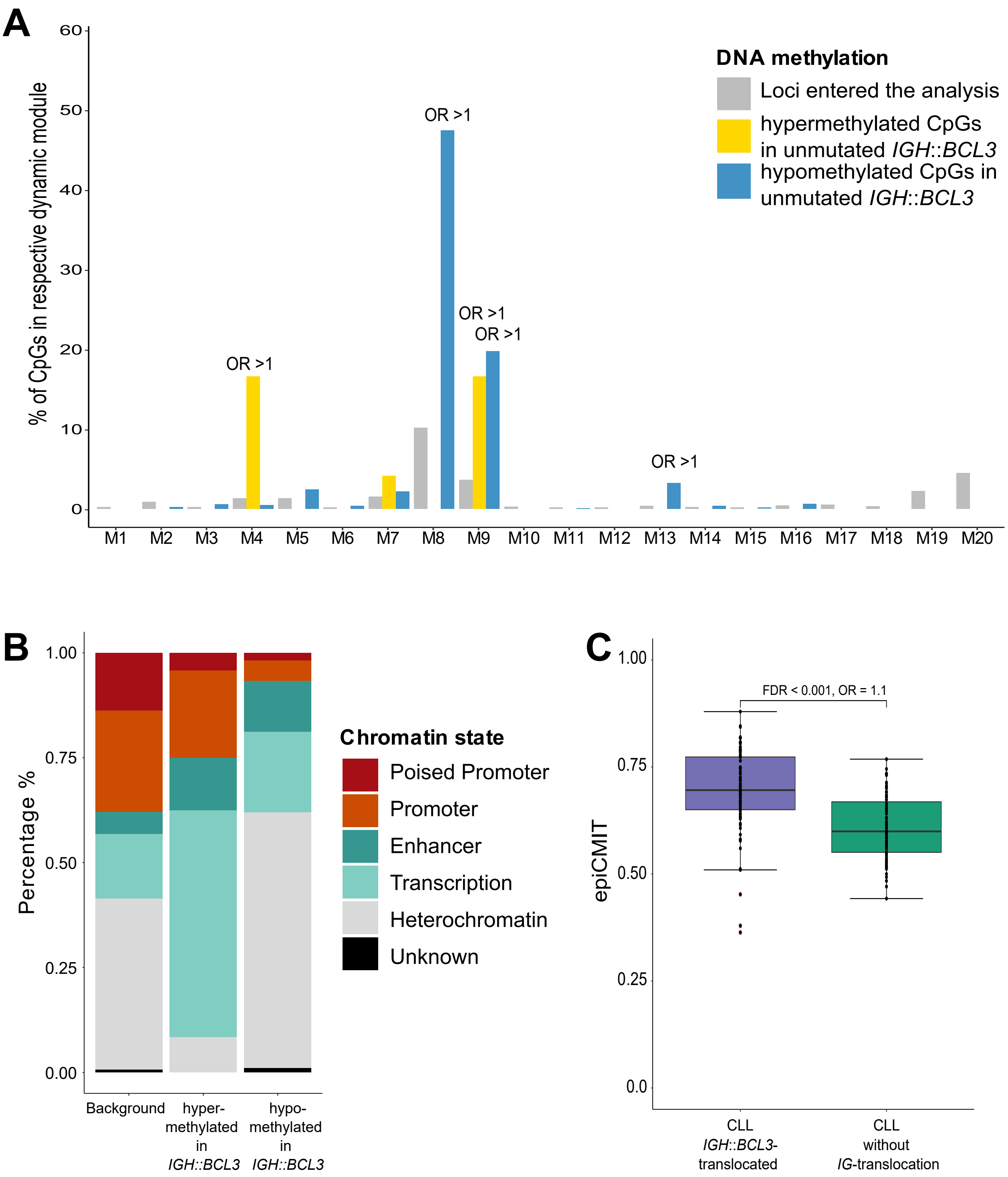

Supplementary Figure 6: DNA methylation profiling of the *IGH*::*BCL3*-translocated CLL samples. A: Barplot of the frequency of Kulis Modules in hyper- and hypomethylated CpGs of the 1985 differential methylated CpGs in unmutated *IGH*::*BCL3*-translocated CLL samples (including follow-up samples). OR = Odds Ratio. **B:** Stacked barplot of the chromatin states of the array background, 25 hypermethylated CpGs and 1960 hypomethylated CpGs in *IGH*::*BCL3* CLL samples (including follow-up samples) compared to unmutated CLL samples without *IG*-translocation (n = 40). **C:** Boxplot showing the significant higher epiCMIT of *IGH*::*BCL3*-translocated CLL samples (without follow-up samples) compared to CLL samples without *IG*-translocation as control (n = 91).

Supplementary Figure 7

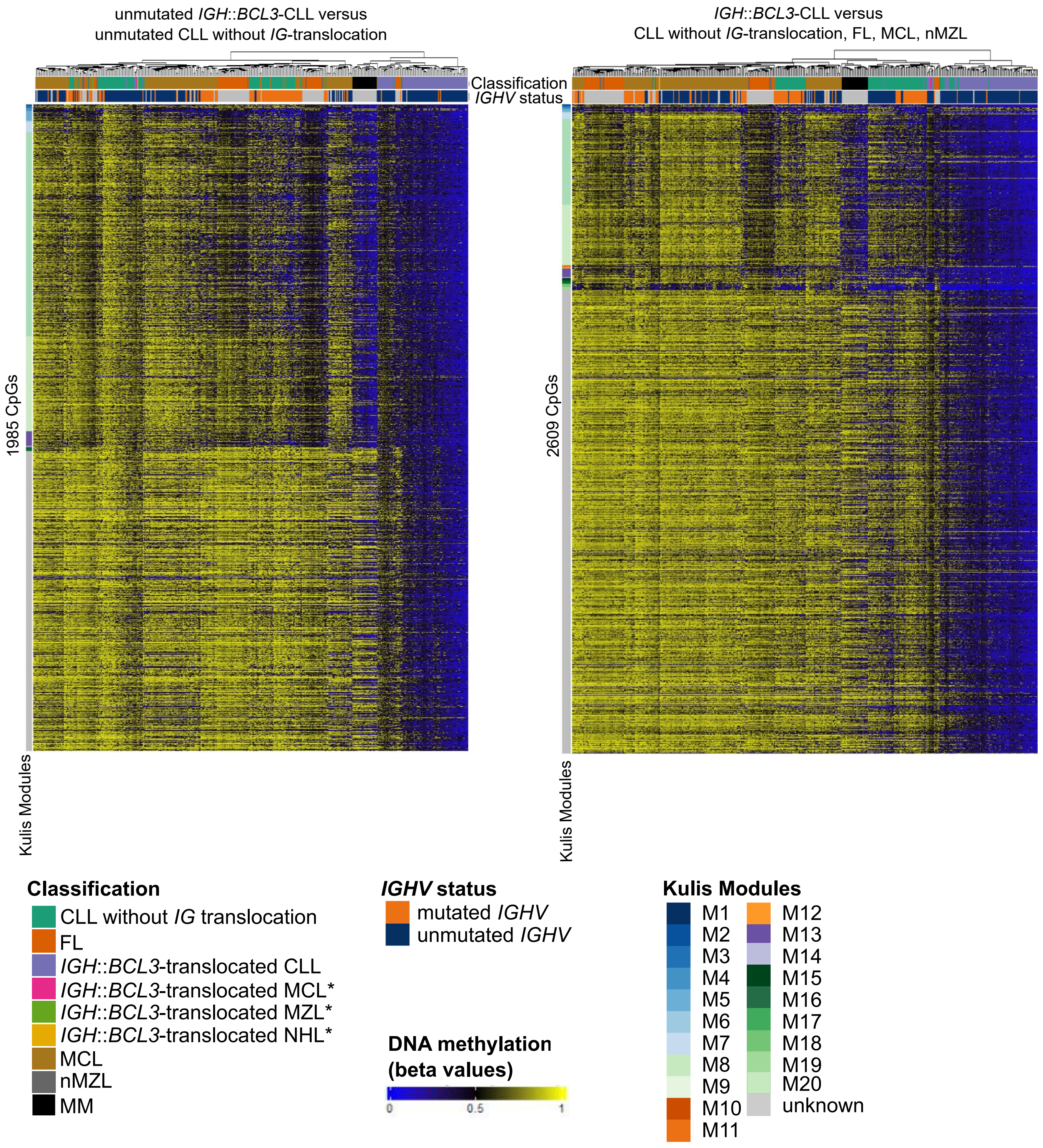

Supplementary Figure 7: DNA methylation profiling of *IGH*::*BCL3*-translocated CLL samples and other B-cell neoplasms including multiple myelomas. Heatmaps of supervised analyses from unmutated *IGH*::*BCL3*-CLL samples (including follow-up samples) and unmutated CLL samples without *IG*-translocation (FDR < 0.01; |Δβ| > 0.3) on the left and from *IGH*::*BCL3*-CLL samples (including follow-up samples) and FL-, nMZL- and MCL-samples (FDR < 0.01; |Δβ| > 0.3) on the right. Additionally, the multiple myeloma (n = 24; MM) are displayed in black. CLL: chronic lymphocytic leukemia, FL: follicular lymphoma, MCL: mantle cell lymphoma, nMZL: nodal marginal zone lymphoma, NHL: Non-Hodgkin Lymphoma, *IG*: immunglobulin. Low DNA methylation values are coloured in blue and high DNA methylation values in yellow. * = non-CLL samples included.

Supplementary Figure 8

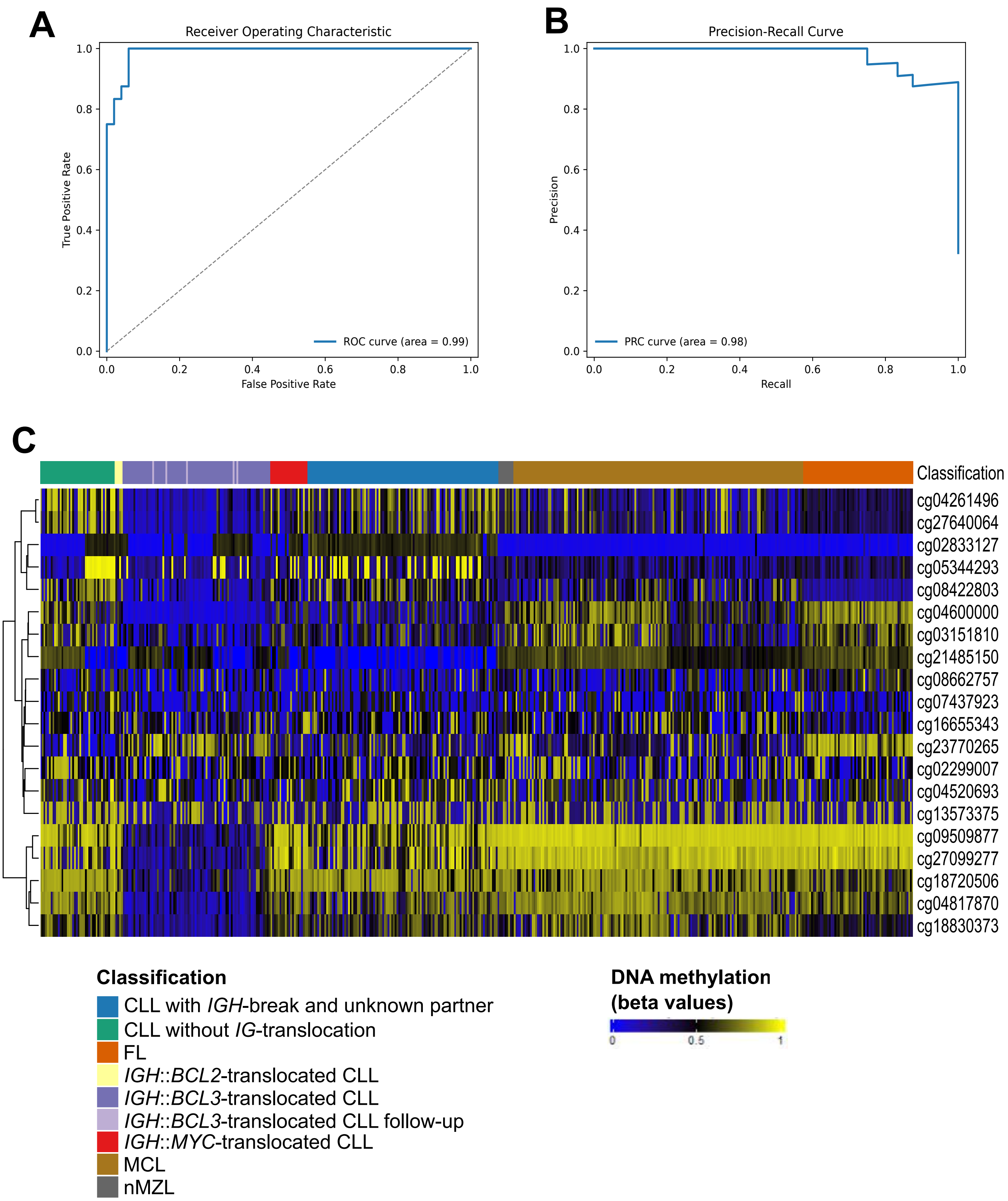

Supplementary Figure 8: Details from the binary *BCL3* classifier. A: Area Under the Receiver Operating Characteristic Curve (AUC-ROC) of the *BCL3* classifier. **B:** Area Under the Precision Recall Curve (AUC-PRC) of the *BCL3* classifier. **C:** Heatmap of the 20 CpGs from the binary *BCL3* classifier in *IGH*::*BCL3*-CLL samples (including follow-up samples), CLL samples with *IGH*-break and unknown partner, CLL samples with *IGH*::*MYC* and CLL samples with *IGH*::*BCL2*, unmutated CLL samples without *IG*-translocation, FL, nMZL and MCL. CLL: chronic lymphocytic leukemia, FL: follicular lymphoma, MCL: mantle cell lymphoma, nMZL: nodal marginal zone lymphoma, *IG*: immunglobulin. Low DNA methylation values are coloured in blue and high DNA methylation values in yellow.

Supplementary Figure 9

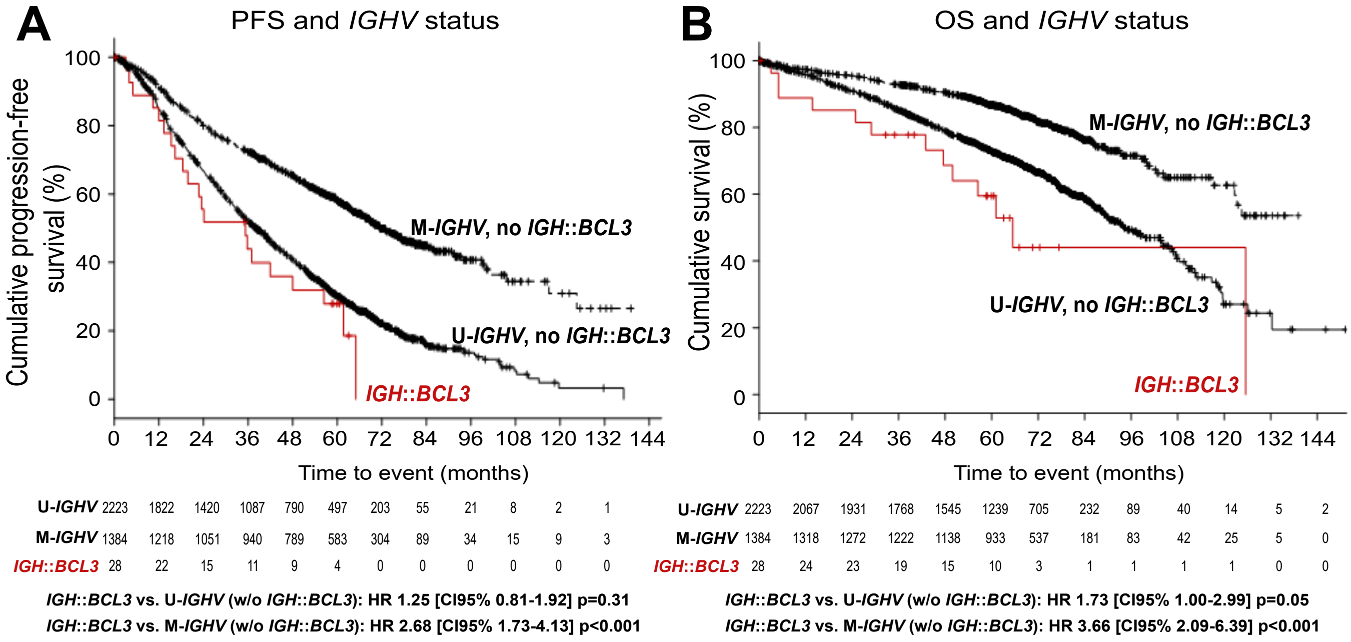

Supplementary Figure 9: Kaplan-Meier estimates of *IGH*::*BCL3*-translocated CLL samples treated in GCLLSG trials compared to CLL without translocation with unmutated and mutated *IGHV* status. Survival plot for progression free survival (PFS) (A) and overall survival (OS) (B) for subgroups according to the *IGHV*-status. HR: Hazard Ratio. CI: Confidence Interval. M-*IGHV*: mutated *IGHV*. U-*IGHV*: unmutated *IGHV*. *IGH*::*BCL3*-translocated CLL samples are depicted in red and CLL samples without *IGH*::*BCL3*-translocation are depicted in black.
